# Proteomic landscape analysis of undifferentiated pleomorphic sarcoma

**DOI:** 10.1186/s13073-026-01626-w

**Published:** 2026-03-20

**Authors:** Biqiang Zheng, Yifei Lu, Bingnan Wang, Wenjun Chai, Mingxia Yan, Zhijian Song, Wangjun Yan

**Affiliations:** 1 Department of Musculoskeletal Oncology, Fudan University Shanghai Cancer Center, Fudan University, Shanghai, 200032 China; 2 Department of Oncology, Shanghai Medical College, Fudan University, Shanghai, 200032 China; 3 Department of Pediatric Surgery, Children’s Hospital of Fudan University, Shanghai, 201102 China; 4 Shanghai Key Laboratory of Birth Defects, Shanghai, 201102 China; 5 Department of Animal Experimental Center, Fudan University Shanghai Cancer Center, Shanghai, 201102 China; 6 Huahai Tianheng Pharmaceutical Research Co., Ltd., Shanghai, 201206 China

**Keywords:** Sarcoma, Proteomics, Recurrence, Macrophages, Lung metastasis

## Abstract

**Background:**

Proteins represent the dynamic downstream products of genes, and have the potential to substantially increase understanding of the complexities of cancer biology. Undifferentiated pleomorphic sarcoma (UPS) is a rare disease, the proteomic landscape of which has not been fully investigated.

**Methods:**

We performed isobaric TMT-based proteomic analysis for 80 UPS tumor and 28 corresponding adjacent non-tumor muscle tissues. The underlying molecular subtypes, therapeutic targets, local recurrence, and lung metastasis were uncovered. The integration of previous single-cell RNA sequencing data with proteomics was further employed to characterize the specific cell type linked to lung metastasis.

**Results:**

Our analyses revealed a catalog of proteins that were dysregulated in UPS. The UPS cohort was stratified into three molecular subtypes S-I, S-II, and S-III. S-I was characterized by the high expression of spliceosome-related proteins and protumor cytokines, and was associated with the lowest overall rate of survival. S-II was characterized with the highest ImmuneScore. S-III with the highest StromaScore had the best outcomes. Protein-based disease classification was better than the recommended AJCC staging system for UPS. PRMT6 protein was overexpressed, and associated with adverse outcomes. PRMT6 inhibitor significantly inhibited tumor growth in two patient-derived xenograft models. Analysis of local recurrence-associated proteins showed that PAWR was negatively associated with UPS recurrence. Additionally, CD163 was a negative predictive marker for lung metastasis. Combined with scRNA-seq data, CD163 + macrophages represented phagocytosis phenotype with high expression of the signature markers (CD163, MRC1, MERTK, and C1QB). Furthermore, CD163 + macrophages were enriched in the endocytosis, phagocytosis, and lysosome compared with CD163- macrophages.

**Conclusions:**

Our study highlights the utility of proteomics for characterizing UPS, providing a framework for understanding the biological basis of its clinical features and paving the way for a new era of proteomics-driven precision medicine in sarcoma research.

**Supplementary Information:**

The online version contains supplementary material available at 10.1186/s13073-026-01626-w.

## Background

Undifferentiated pleomorphic sarcoma (UPS), formerly known as malignant fibrous histiocytoma (MFH), is one of the most frequent soft tissue sarcomas (STS) with diffuse pleomorphism and no identifiable line of differentiation [1]. UPS exhibits highly aggressive phenotype with a local recurrence rate ranging between 19% and 31%, and a metastatic rate of 31% to 35% [1, 2]. The current treatments for UPS are wide resection, radiotherapy, chemotherapy, and immunotherapy; however, therapeutic efficacy is not satisfactory, with the five-year survival rate ranging between 30% and 50% [3, 4]. Therefore, dissecting the molecular characteristics of UPS is critical for understanding its inherent biology and the development and application of new treatment strategies to improve patient survival.

Efforts have been made to study the genomic and transcriptomic landscapes of UPS to provide insight into the tumor biology [5–7]. However, characterizing genetic changes alone is insufficient to interpret the disease occurrence and development. Proteins represent the dynamic downstream products of genes, and have the potential to substantially increase understanding of the complexities of cancer biology. A dysregulated proteome is an essential contributor in carcinogenesis [8–12]. Large-scale, mass spectrometry (MS)-based proteomics have identified novel mechanisms, molecular subtypes and therapeutic targets for patients with esophageal [8], pancreatic [9], liver [10], colon [11], and breast [12] cancers. Proteomic profiling of pan-STS identifies molecular subtypes with distinct biological features and survival outcomes [13, 14]. However, proteomic studies of UPS biology are at a relatively early stage. The proteomic landscape of UPS has not been characterized in a large cohort of patients, and the underlying biology, molecular subtypes, prognostic biomarkers, and therapeutic targets based on proteomics have not been fully elucidated for UPS.

In this study, we performed proteomic analysis of UPS from 80 cases. Integrating the proteomic dataset with clinical data and our previous scRNA-seq data [7], we demonstrated the utility of proteomics resource in uncovering the disease characteristics, molecular subtypes, therapeutic targets, local recurrence, and lung metastasis. Our findings provide new insights into UPS biology and assist patient stratification and therapeutic development.

## Methods

### Patients

Fresh specimens were collected at the time of surgical resection between 2010 and 2019, and were immediately frozen in liquid nitrogen and stored at -80℃ in the tissue bank of Fudan University Shanghai Cancer Center. Patients were histopathologically diagnosed with UPS according to the World Health Organization (WHO) classification, by at least two pathologists. Written informed consent was obtained from participants, and the study was approved by the Clinical Research Ethics Committee of the Fudan University Shanghai Cancer Center (Number: 050432-4-2108). Fresh tissue samples from UPS tumors (*n* = 80) and their adjacent non-tumor tissues (*n* = 28) were subjected to proteomic profiling. From these samples, we established the proteomic cohort, which served as the discovery cohort in this study. The last follow-up date for the patients with UPS was August 2023; the shortest follow-up time was 44 months while the longest follow-up time was 158 months. Patient demographics (age and gender), clinicopathological features (tumor location and disease stage), and clinical outcomes (recurrence, metastasis, and survival time) were collected for analysis.

An independent cohort of 75 cases was used as the validation cohort. The surgical period spanned from 2006 to 2015. Tumor specimens were preserved in paraffin blocks and were sectioned into 5-micrometer slices when needed. The last follow-up for patients was conducted in 2023. The validation cohort was used to detect the expression of PRMT6, PAWR, and CD163 proteins by immunohistochemical staining. Clinical parameters were collected for analysis: gender, age, tumor location, disease stage, recurrence, metastasis, and survival time.

### Protein extraction and digestion

For protein extraction [15], SDS lysis buffer was added into the powdered tissues, and sonicated at 15% amplitude, 5 s on and 5 s off, with a total working time of 2 min. The proteins were then heated at 95 °C for 5 min. After centrifugation at 12,000 × g for 10 min at 4 °C, the supernatant containing total protein was collected. Protein concentration was determined using the bicinchoninic acid (BCA) assay (Thermo Fisher Scientific, Waltham, MA, USA) according to the manufacturer’s protocol.

Each sample was processed using the Filter-Aided Sample Preparation (FASP) method. Briefly, proteins were loaded onto Microcon-30 kDa centrifugal filter units (Millipore, Burlington, MA, USA). SDS was removed by washing with 8 M urea in 0.1 M Tris-HCl, pH 8.5 (UA buffer). After reduction with 10 mM dithiothreitol (DTT) in UA buffer for 30 min, alkylation was performed by adding 50 mM iodoacetamide (IAA) in UA buffer for 20 min in the dark, at room temperature. After washing with UA buffer and subsequently with 50 mM ammonium bicarbonate, proteins were digested overnight at 37 °C with sequencing-grade modified trypsin (Promega, Madison, WI, USA) at an enzyme-to-protein ratio of [1:50 (w/w)]. After digestion, peptides were collected by centrifugation. Combined peptide eluates were then desalted using C18 Sep-Pak cartridges (Waters, Milford, MA, USA) according to the manufacturer’s instructions. Finally, the desalted peptides were lyophilized to dryness and stored at -80 °C. An internal reference sample was created by pooling an aliquot from 80 UPS tissues and 28 nontumorous muscle tissues. The reference sample was also prepared by FASP.

### Tandem mass tag (TMT) 10-plex labeling

Lyophilized peptides derived from 80 UPS and 28 adjacent nontumorous muscle tissue samples were dissolved in 100 mM TEAB. Peptide concentrations were determined using a peptide BCA assay. For TMT labeling, 25 µg of peptides from each sample was labeled with each 20 µL of reconstituted TMT Label Reagent (Thermo Scientific) following the manufacturer’s protocol. Labeling reactions were performed for 1 h at room temperature. The internal reference sample was labeled with one TMT channel and included in each of the TMT 10-plex sets to facilitate normalization and comparison across batches. After labeling, the reactions were quenched with 5% hydroxylamine. TMT-labeled samples within each 10-plex set were then combined, desalted using C18 Sep-Pak cartridges, and lyophilized.

### Liquid chromatography-tandem mass spectrometry (LC-MS/MS) analysis

TMT-labeled peptide mixtures were dissolved in buffer A (0.1% formic acid). LC-MS/MS analysis was performed on an Easy-nLC 1200 liquid chromatography (LC) system (Thermo Fisher Scientific) coupled online to a Q Exactive HFX mass spectrometer (Thermo Fisher Scientific) via a nano-electrospray ion source. Peptides were loaded onto a trap column (Acclaim PepMap 100, 100 μm × 2 cm C18, 5 μm, Thermo Fisher Scientific) and subsequently separated on an analytical column (EASY C18 column, 75 μm × 50 cm, 2 μm) with a linear gradient ranging from 9 to 32% acetonitrile in 100 min, followed by a linear increase to 50% Solvent B (0.1% formic acid in 80% acetonitrile) in 20 min, at a flow rate of 300 nL/min. The mass spectrometer was operated in data-dependent acquisition mode. Full MS scans were acquired in the Orbitrap analyzer over a mass range of 300 to 1800 m/z with a resolution of 70,000 at 200 m/z. The automatic gain control (AGC) target was set to 1e6 with a maximum injection time (IT) of 50 ms. Dynamic exclusion was set to 30 s. Only spectra with a charge state of 2–6 were selected for fragmentation by higher-energy collision dissociation using a normalized collision energy of 32%. The MS2 spectra were acquired in the Orbitrap, at a resolution of 17,500 at 200 m/z, an AGC target of 1e5, and a maximum IT of 105 ms.

### Protein identification and quantification

All mass spectrometric data were analyzed using MaxQuant software (version 1.6.1.0). The MS/MS spectra were searched against the human Swiss-Prot database. For protein identification and quantification, TMT 10-plex based MS2 reporter ion quantification was chosen. The reporter mass tolerance was set at 0.003 Da. The precursor intensity fraction (PIF) filter value was set at 0.5 to reduce the interference of precursor co-fragmentation. Search parameters were set as follows: enzyme specificity was Trypsin, allowing up to 2 missed cleavages. Carbamidomethylation of cysteine was set as a fixed modification. Oxidation of methionine, protein N-terminal acetylation, lysine acetylation, and deamidation of asparagine and glutamine (NQ) were set as variable modifications. A maximum of 5 modifications per peptide were allowed. The precursor mass tolerance was set to 20 ppm for the first search and 4.5 ppm for the main search. The minimal peptide length was set at 7 amino acids.

Peptide-spectrum matches (PSMs), peptides, and proteins were filtered to achieve a false discovery rate (FDR) of < 0.01 at all levels (peptide, protein). For TMT quantification, the purities of TMT labeling channels were corrected according to the kit LOT number. Protein quantification was based on TMT reporter ion intensities. Only unique and razor peptides were considered for quantification. Proteins identified by at least one unique or razor peptide were retained for further analysis.

### Data filtering, normalization, and missing value imputation

Proteins with > 50% missing values across all samples were excluded from the dataset to ensure robustness in downstream analyses. The remaining data were then normalized using the median centering method across total proteins to correct for potential sample loading differences. For this, reporter ion intensities were typically log-transformed, and median-centered across all samples, resulting in values centered around zero. Normalized protein identifiers (SwissProt IDs) were converted to Human Genome Nomenclature Committee (HGNC) HUGO symbols using resources provided by HGNC (https://www.genenames.org). Following normalization, K-nearest neighbor (k-NN) imputation was applied to impute the remaining missing values in the protein quantification matrix. This imputation was performed using the pamr package in R.

### Batch effect assessment and data quality control

To assess potential batch effects, for instance, those arising from different TMT multiplexes, unsupervised principal component analysis (PCA) was performed on the normalized and imputed proteomic data. PCA was conducted using prcomp functions in R. Visualization of the principal components (PC1 vs. PC2) was employed to examine sample clustering patterns, particularly to confirm expected biological separations (such as tumor from normal samples) and to verify the absence of significant clustering by TMT batch.

### Proteomic subtype identification

To identify distinct proteomic subtypes of UPS, consensus clustering was performed on the normalized and imputed protein abundance data from tumor samples (*n* = 80). For subgrouping, the analysis utilized the top 25% most variable proteins, selected based on their high variance across these tumor samples. Consensus clustering was implemented on these selected proteins using the Consensus Cluster Plus R package. The following detailed settings were used for clustering: number of repetitions = 1,000 bootstraps; pItem = 0.8 (resampling 80% of samples); pFeature = 0.8 (resampling 80% of proteins); and k-means clustering with exploration up to 6 clusters (k).

### Gene ontology and pathway enrichment analysis for proteomic subtypes

To elucidate the biological functions characterizing each proteomic subtype, we first defined their protein signatures. This was achieved by applying k-means clustering (k = 3) to the expression profiles of the top 25% most variable proteins. Each resulting protein cluster was considered the signature for its corresponding subtype. For each subtype, lists of candidate proteins were subjected to Gene Ontology (GO) enrichment analysis (Biological Process, Molecular Function, Cellular Component) or KEGG pathway enrichment analysis. These analyses were performed using clusterProfiler R package.

### Tumor microenvironment analysis

Tumor microenvironment (TME) based on the proteomic subtyping was analyzed through a two-step analytical pipeline. First, we employed the Molecular Functional Portraits (MFP) pipeline [15](https://github.com/BostonGene/MFP) to perform feature extraction on our proteomic data. This approach quantifies the activity of 29 curated, TME-related functional pathways by calculating single-sample Gene Set Enrichment Analysis (ssGSEA) scores for each sample. Subsequently, the resulting signature matrix of ssGSEA scores served as the input for unsupervised consensus clustering; ConsensusClusterPlus R package was used to characterize the proteomic subtypes S-I, S-II, and S-III.

The relative abundance of cell types, including monocytes, macrophages, fibroblasts, and endothelial cells, was estimated for each tumor sample. This estimation was performed using the computational deconvolution algorithm xCell [16](https://xcell.ucsf.edu/). Differences in the estimated cell fractions among the proteomic subtypes were assessed using the Kruskal-Wallis test, followed by pairwise comparisons with the Wilcoxon rank-sum test. The MHC score was represented by the normalized enrichment score (NES) of the corresponding KEGG gene set, as calculated previously via single-sample Gene Set Enrichment Analysis (ssGSEA).

### Analysis of the cancer genome atlas (TCGA) UPS data

We first identified the genes encoding the defining protein markers of our three proteomic subtypes. We then mapped their expression to the TCGA UPS cohort and finally re-classified the samples using a k-means–based consensus clustering algorithm on this signature’s expression data, thereby independently reconstructing the subtype structure. The prognostic relevance of this TCGA-based subtype classification was evaluated using clinical follow-up data from the TCGA-UPS cohort. Overall survival (OS) curves were generated using the Kaplan-Meier method, and differences between the resulting subtypes were assessed using the log-rank test.

### Proteomic analysis of clinical outcomes

To identify protein expression patterns associated with clinical outcomes (recurrence and lung metastasis) in our patient cohort, a differential proteomic analysis was performed. Patients were stratified into groups based on recurrence status (recurrent vs. non-recurrent) and lung metastasis status (lung metastasis vs. no lung metastasis). Differential protein expression between these groups was analyzed using the Wilcoxon rank-sum test on the normalized and imputed protein abundance data. Proteins with *P* < 0.05 and a log2 FC > 0.263 were considered significantly associated with either recurrence or lung metastasis.

### Dimension reduction and clustering for UPS scRNA-seq data

UPS scRNA-seq data [7] were processed using the Seurat package. The gene expression matrix was normalized using the NormalizeData function. Unique molecular identifiers (UMIs) of each gene were divided based on the sum of all UMIs of each cell, multiplied by 10,000, and transformed to a log scale. The FindVariableFeatures function was used to identify highly variable genes. Data were scaled prior to Principal component analysis (PCA). Clustering of cells was performed using FindNeighbors and FindClusters. Cell embeddings on two dimensions were calculated using RunUMAP. Differential expression of genes was visualized on Uniform Manifold Approximation and Projection (UMAP).

### Analysis of gene correlations at single cell levels

Correlation analysis was performed using Pearson correlation, with statistical significance assessed by a t-test. The results were visualized using the ggscatterstats function from the ggstatsplot package.

### GO enrichment analysis of CD163 + macrophages

Enrichment analysis for CD163 + macrophages was performed using ClusterProfiler package. Top 50 differentially expressed genes (DEGs) of CD163 + macrophage vs. CD163- macrophage were selected as input genes. We used gene sets from the GO database to determine functions of cell types.

### Cell–cell interaction analysis

Cell-cell interactions were analyzed by CellphoneDB. Ligand-receptor interaction scores were calculated. Significant and representative interactions were visualized by plot_cpdb function provided in ktplot package.

### Immunostaining assays

Immunohistochemistry (IHC) and immunofluorescence (IF) staining were performed as previously described [7]. The following antibodies were used: PRMT6 (Abcam, ab72205), PAWR (Proteintech, 20688-1-AP), CD163(Abcam, ab182422), DLK1 (Proteintech, 10636-1-AP), and COL3A1 (Abcam, ab7778). Two pathologists conducted IHC analysis to evaluate the staining patterns of each sample. The PRMT6 staining scores were evaluated [17] and the percentage of positively stained cells were assigned scores as follows: 1 (< 10%), 2 (10–50%), 3 (50–75%), and 4 (> 75%). Staining intensity was scored on a scale of 0–3: 0 for no staining, 1 for light yellow, 2 for yellow-brown, and 3 for brown. The staining indices were determined by multiplying the percent score with the intensity score. For PAWR, a score was assigned according to the intensity and percentage of positively stained cells [18], 0 to + 1, no staining or staining in ≤ 10% of cells; +2, weak to-moderate staining in > 10% of cells and + 3, strong staining in > 10% of cells. A score of ≥ 2 was considered positive. Macrophage biomarker (CD163) was scored by counting the number of positive-staining macrophages/mm^2^.

### PDX model

Animal experiments were carried out according to the protocol approved by the Institutional Animal Care and Use Committee of the Fudan University Shanghai Cancer Center (Number: FUSCC-IACUC-2022188). UPS PDX model was established as described previously [7]. Animals were housed under specific pathogen-free conditions with a 12:12-hour light-dark cycle, an ambient temperature of 22 ± 1 °C, and a relative humidity of 50 ± 10%. Tumor samples were implanted subcutaneously in the right flank and allowed to develop to an average volume of 60 mm^3^. The mice were randomized to either the control (normal saline) group or the EPZ (at a dose of 10 mg/kg) group using complete randomization [19]. All mice received drugs or normal saline daily via subcutaneous administration. Tumor sizes were measured twice a week using a caliper, and tumor volumes were calculated using the following formula: volume = 1/2 length × width^2^. All mice were euthanized by carbon dioxide asphyxiation in a dedicated euthanasia chamber, and tumors were harvested. Student’s t-test was used to compare tumor volume between the two groups at 6 weeks. The effect size was represented by Cohen’s d, calculated as (control group mean - experimental group mean) / pooled standard deviation.

### Statistical analysis

Standard statistical tests were used to analyze the associations between clinical information and proteomic data. For continuous data, Student’s t-test or the Wilcoxon rank-sum test was used for two-group comparisons, while one-way ANOVA or the Kruskal-Wallis test was applied for comparisons of more than two groups. For categorical data, Fisher’s exact test was employed. Survival analyses were conducted using the log-rank test. Cox regression analysis was performed using R software (version 4.3). All statistical tests were two-sided, and a p-value < 0.05 was considered statistically significant. Correlations between two datasets were calculated using either Pearson’s or Spearman’s correlation coefficient. Significance levels are denoted as follows: *p < 0.05, **p < 0.01, and ***p < 0.001.

## Results

### Proteomic analysis of UPS

**Fig. 1:**
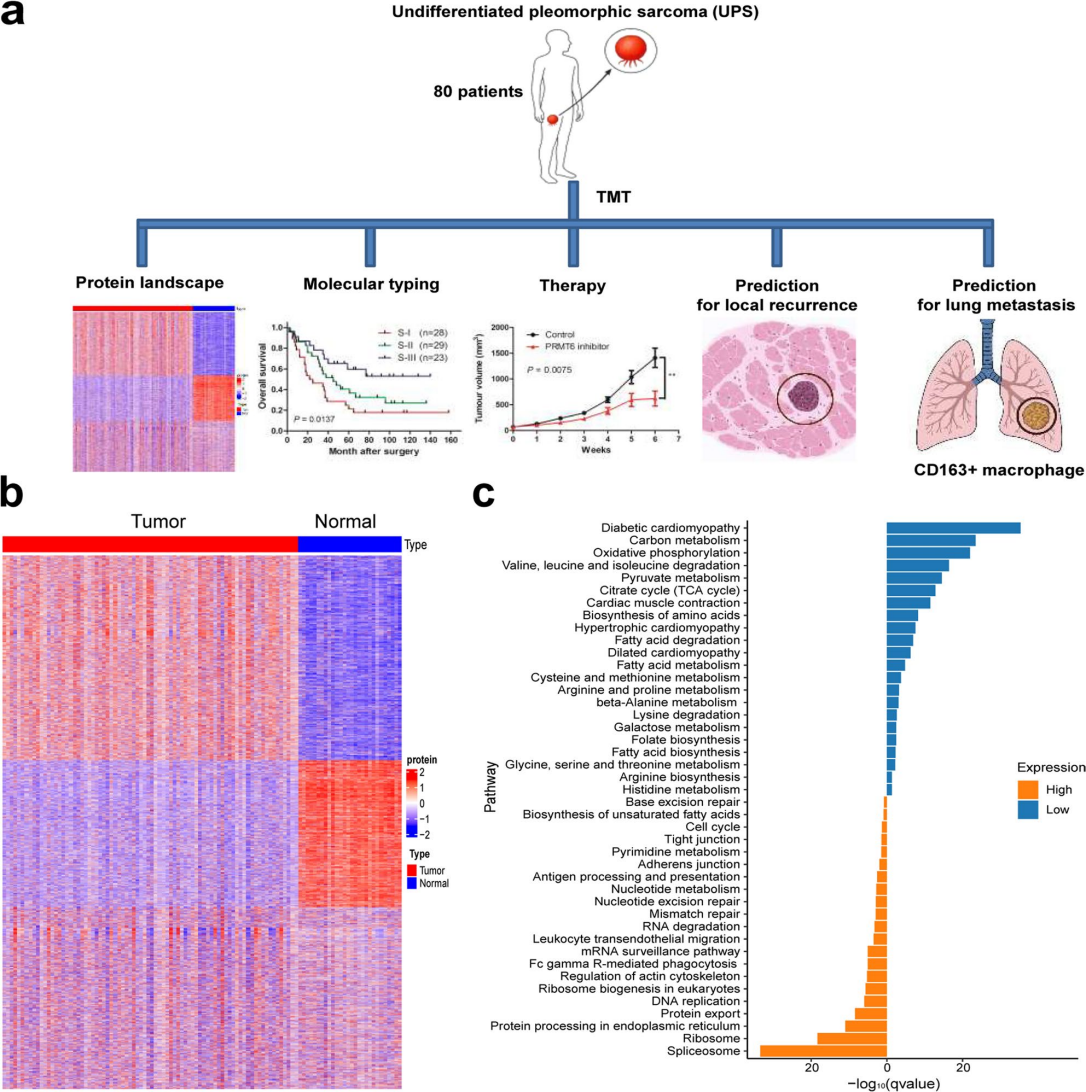
Proteomic analysis of undifferentiated pleomorphic sarcoma (UPS).** a** workflow of the UPS proteomic study. **b** Hierarchical clustering of 80 UPS tumor and 28 corresponding adjacent non-tumor muscle tissues. **c** Dysregulated KEGG pathways in UPS. Orange bars indicate pathways enriched with proteins upregulated in UPS. Blue bars indicate pathways enriched with proteins downregulated in UPS

We performed isobaric TMT-based proteomic analysis for 80 UPS tumor and 28 corresponding adjacent non-tumor muscle tissues (Fig. 1a and Table S1), which identified a total of 11,840 proteins. Among them, 5,861 and 3,426 proteins were quantifiable in at least half of the samples and all samples, respectively. PCA revealed that tumors were well separated from non-tumors (Fig. S1). Additionally, no batch effects were observed among groups (Fig. S1).

We next identified proteins that were differentially expressed between tumor and non-tumor samples. Among the 5,861 proteins that were quantifiable (Table S2), 2,356 (40.2%) and 1,939 (33.1%) were significantly upregulated and downregulated, respectively, in tumors compared to paired nontumors (Table S3). Of these, 1,221 proteins exhibited an FC larger than 1.5, with 489 and 732 being up-regulated and downregulated in tumors, respectively (Table S3). The five most up-regulated proteins were PTMA, PRMT6, SPIRE1, CLCNKA, and MARCKS (Table S3). These were associated with angiogenesis [20], cellular transport processes [21], and tumor progression [19, 22]. The proteins (MYH4, TNNT3, CASQ1, MYH7, MYH2, and MYL2) that are exceptionally enriched in muscle cells were obviously down-regulated in UPS (Table S3).

UPS had an obvious different proteomic landscape compared with the corresponding adjacent non-tumor tissues (Fig. 1b). Pathway enrichment analysis revealed that RNA splicing, protein synthesis, processing and export, DNA replication, mismatch repair, nucleotide excision repair, FCγR-mediated phagocytosis, antigen processing and presentation, and nucleotide metabolism were over-represented in UPS (Fig. 1c and Table S4), whereas, muscle contraction and many kinds of metabolism-related pathways were over-represented in adjacent non-tumor muscle tissues (Fig. 1c and Table S5). Interestingly, compared with non-tumor muscle tissues, UPS showed low metabolism in carbon metabolism, oxidative phosphorylation, pyruvate metabolism, citrate cycle, and biosynthesis of amino acids, folate, fatty acids, and arginine.

### Molecular subtypes of UPS defined by proteomic analysis are associated with prognosis

**Fig. 2:**
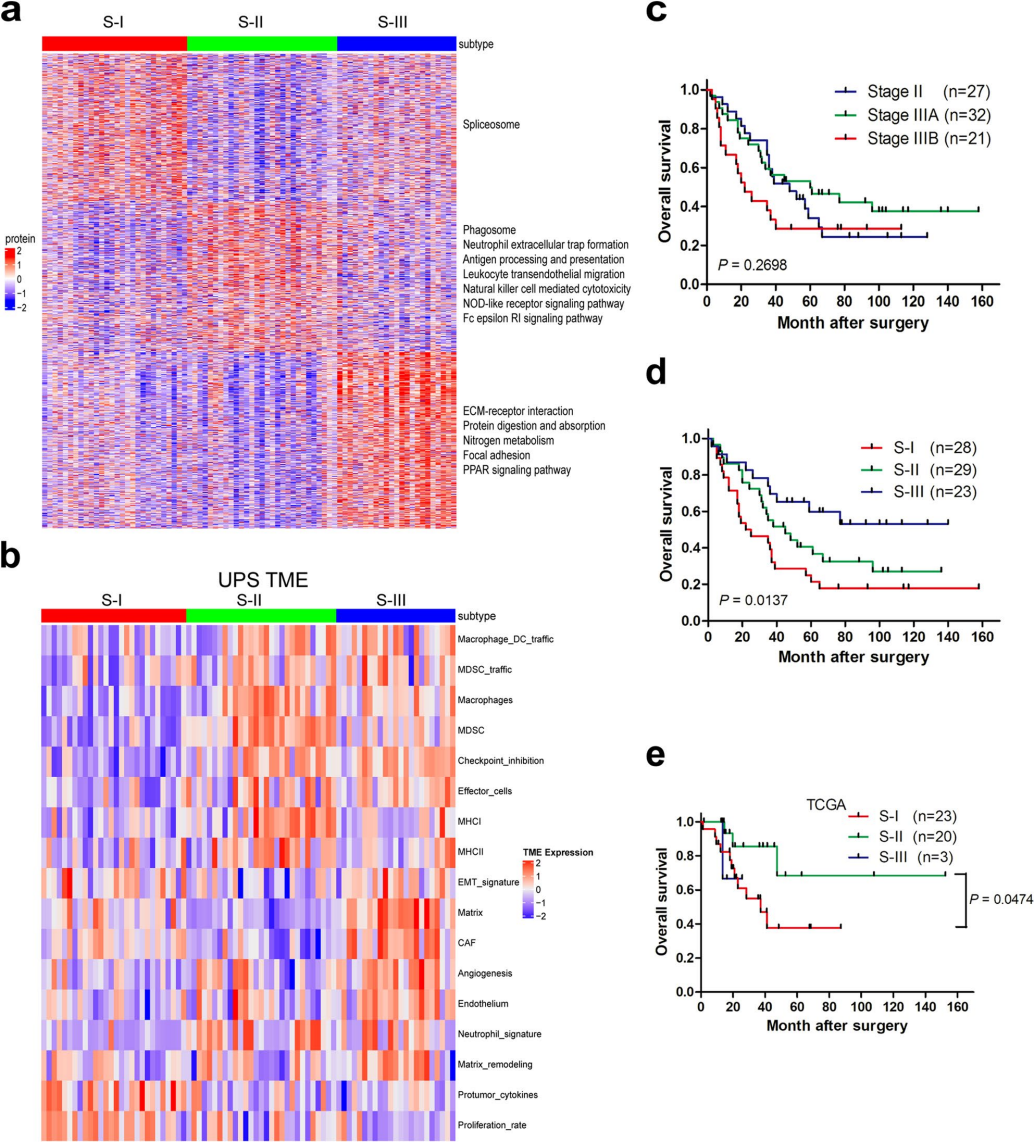
Proteome-based stratification of UPS reveals three subtypes related to different tumor microenvironments (TMEs) and prognoses.** a** Consensus-clustering analysis of proteomic profiling identifies three proteomic subtypes. The heat map depicts the relative abundance of signature proteins. Representative KEGG related to these signature proteins are denoted on the right. **b** Analysis of TME for proteomic subtypes S-I, S-II, and S-III. **c** Prognostic stratification of UPS (*n* = 80) according to current TNM staging system. **d** Prognostic stratification of UPS (*n* = 80) according to proteomic subtyping. **e** Prognostic stratification of UPS (*n* = 46) from clustering of TCGA UPS mRNA data with our proteomic signature

UPS is a heterogeneous malignancy, which supports our stratification analysis. Using k-means-based consensus clustering, we identified three major proteomic subtypes: 28, 29, and 23 cases were identified as belonging to subtypes S-I, S-II, and S-III, respectively (Fig. 2a). Interestingly, different subtypes were accompanied by distinct characteristics and KEGG pathways (Table S6 and S7). Subtype S-I was characterized by the high expression of spliceosome-related proteins, such as PQBP1, FUS, TRA2B, HNRNPU, SRSF6, DHX38, SF3B5, CWC15, and PPIE. In addition, proliferative protein MKI-67 was also upregulated in the subtype S-I. Subtype S-II was characterized by immune infiltration (monocytes and macrophages). Inflammatory pathways such as phagosome, neutrophil extracellular trap formation, antigen processing and presentation, leukocyte transendothelial migration, hematopoietic cell lineage, natural killer cell mediated cytotoxicity, NOD-like receptor signaling pathway, and Fc epsilon RI signaling pathway were specially enriched in subtype S-II; subtype S-III, with abundant stromal proteins (COL1A1, COL1A2, COL3A1, and FN1), represented extracellular matrix (ECM) type, and ECM-receptor interaction, protein digestion and absorption, nitrogen metabolism; focal adhesion and PPAR signaling pathway were specifically high in S-III. The TME was further analyzed based on proteomic subtyping. Subtype S-I displayed high expression of protumor cytokines and proliferation rate (Fig. 2b). Subtype S-II characterized by the highest ImmuneScore was enriched with monocytes, macrophages, MHC I, and MHC II (Fig. 2b and Fig. S2). Subtype S-III with the highest StromaScore showed the largest numbers of fibroblasts and endothelial cells (Fig. 2b and Fig. S2).

The current TNM staging system showed limited clinical value in prognostic stratification of the 80 UPS (Fig. 2c, *P* = 0.2698). Interestingly, the proteomic subgroups significantly differed in survival (Fig. 2d, *P* = 0.0137), subtype S-I showed the worst survival, subtype S-III had the best survival, patients assigned to S-II appeared to have an intermediate prognosis, indicating that proteomic subgroups stratified these patients better than TNM staging-based subgroups. Clustering of TCGA UPS mRNA data (*n* = 46) with our proteomic signature resulted in three subgroups, S-III had only three patients. In accordance with our results, S-I (*n* = 23) showed worse prognosis than S-II (*n* = 20, Fig. 2e, *P* = 0.0474).

### PRMT6 is a potential target for treating UPS

**Fig. 3:**
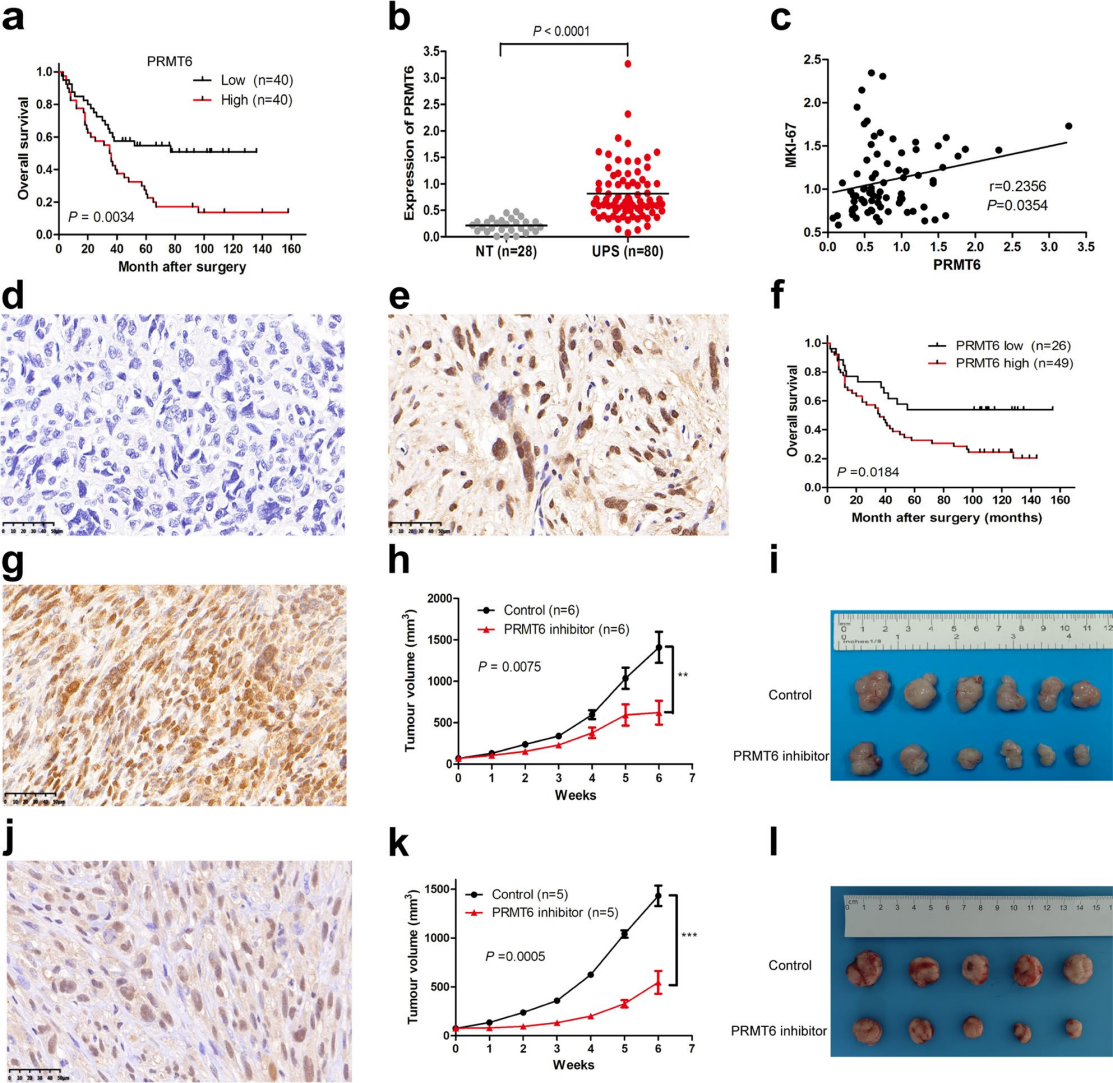
PRMT6 is a potential target for treating UPS. **a** Kaplan-Meier curves of overall survival for PRMT6 expression levels in the proteomic cohort. **b** Comparison of PRMT6 protein expression levels between 28 corresponding adjacent non-tumor (NT) muscle tissues and 80 UPS tumors in the proteomic cohort. **c** Correlation of PRMT6 with Ki-67 proliferation index. **d** Representative negative PRMT6 expression by immunohistochemical staining in UPS. **e** Representative positive PRMT6 expression in UPS. **f** Kaplan-Meier curves of overall survival for PRMT6 expression in an independent validation cohort (*n* = 75), as detected by immunohistochemical staining. **g** High expression of PRMT6 protein in a UPS PDX model. **h**,** i** In vivo study of the effects of RMT6 inhibitor (EPZ020411) on the UPS PDX model. Tumor volume data at the indicated time points are presented as mean ± SEM (*n* = 6 per group). **j** High expression of PRMT6 protein in the second UPS PDX model. **k**,** l** In vivo study of the effects of RMT6 inhibitor (EPZ020411) on the second UPS PDX model. Tumor volume data at the indicated time points are presented as mean ± SEM (*n* = 5 per group)

To seek therapeutic strategies for patients with UPS, we focused on the overexpressed proteins in UPS. The top 5 up-regulated proteins were PTMA, PRMT6, SPIRE1, CLCNKA, and MARCKS (Table S3). However, only PTMA and PRMT6 were negatively associated with unfavorable prognosis (Fig. 3a and Fig. S3). The role of PTMA and PRMT6 in UPS has not been reported. Compared with PTMA, there is a selective inhibitor (EPZ020411) for PRMT6. In the multivariable Cox regression analysis, PRMT6 was identified as an independent predictor of adverse prognosis (HR = 3.32, *P* = 0.0005, Table S8). PRMT6 is frequently elevated in human cancers, and its expression contributes to tumor malignancy [17, 19, 23]. Therefore, we selected PRMT6 for further analysis. The PRMT6 protein was overexpressed by almost 4-fold in UPS compared with their corresponding nontumorous tissues (Fig. 3b). Interestingly, we found PRMT6 was positively correlated with Ki-67 proliferation index (Fig. 3c; *r* = 0.2356, *P* = 0.0354), and was highly expressed in subtype S-I (Fig. S4). To validate the clinical significance of PRMT6, we employed an independent validation cohort (*n* = 75) for which detailed clinical information is provided in Table S9. In this cohort, PRMT6 expression was assessed by immunohistochemical staining (Fig. 3d and e). Positive PRMT6 expression was detected in 49 of 75 patients and was significantly associated with adverse clinical outcomes (Fig. 3f, *P* = 0.0184).

UPS is a rare disease; there is no available cell line for UPS from Cell Bank of Chinese Academy of Sciences or American Type Culture Collection (ATCC). Therefore, we established two PDX models from two patients with UPS. High expression of PRMT6 protein was observed in the two PDX models (Fig. 3g and j). We further tested the effectiveness of PRMT6 inhibitor (EPZ020411) in the two PDX models. EPZ020411 significantly inhibited tumor growth in both PDX models (Fig. 3h and i, *P* = 0.0075; Fig. 3k and l, *P* = 0.0005). The inhibitory effect, as quantified by Cohen’s d, was substantial, with values of 1.93 and 3.58 for the comparisons shown in Fig. 3i and l, respectively. Furthermore, the treatment had no effect on body weight (Fig. S5), and no obvious toxicity was observed compared to the control groups.

### Dissection of local recurrence-associated proteins

**Fig. 4:**
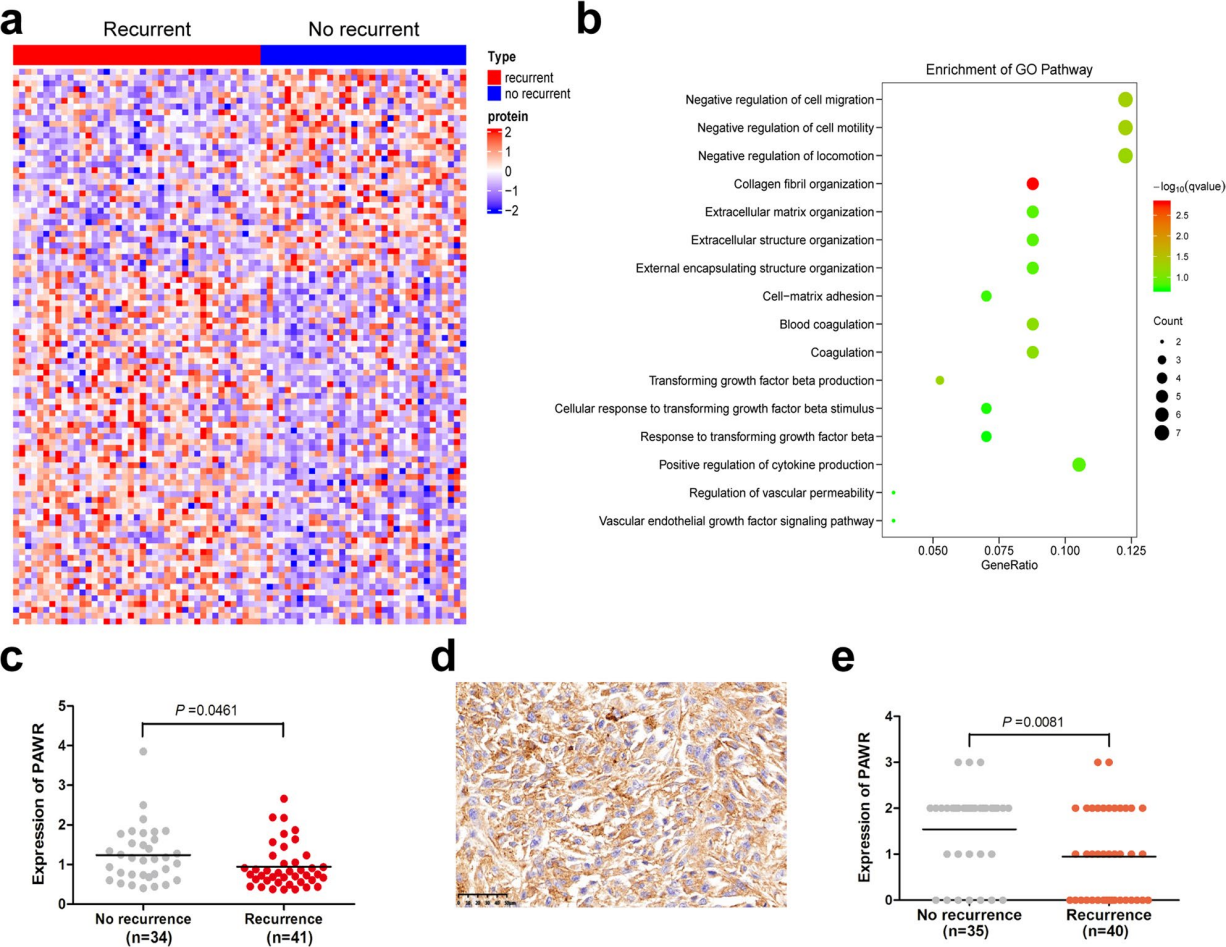
Dissection of local recurrence-associated proteins.** a** Consensus-clustering analysis of proteomic profiling with local recurrence. **b** Gene ontology (GO) analysis of local recurrence. **c** Comparison of PAWR protein expression levels between no local recurrence group and recurrence group in the proteomic cohort. **d** Representative positive PAWR expression by immunohistochemical staining in UPS. **e** Comparison of PAWR protein expression levels between no local recurrence group and recurrence group, in the independent validation cohort, based on immunostaining scores

Local recurrence is a critical challenge in the management of UPS, which has a high local recurrence rate of 19% -31% [1, 2]. In the 80 resected UPS, except for five cases with a positive margin, 41 cases showed local recurrence after surgery, whereas 34 cases had no local recurrence in the long-term follow-up (44–158 months). We compared the proteomics data between patients with relapse (*n* = 41) and patients with no relapse (*n* = 34). Proteins associated with local recurrence are shown in Table S10. Hierarchical clustering revealed that recurrent UPS had a different proteomics profile compared to that for no recurrent UPS (Fig. 4a). GO analysis revealed that biological processes such as negative regulation of cell migration, negative regulation of cell motility, and collagen fibril organization were enriched in tumors associated with UPS relapse (Fig. 4b and Table S11). Our results showed that 60 proteins were positively associated with local recurrence (Table S10). Among these, COL14A1 was the top protein, which interacts with extracellular matrix structural constituent, and is considered as an oncoprotein in tumor progression [24]. We found 36 proteins inversely correlated with local relapse (Table S10). Of them, PAWR was the most downregulated protein in recurrent UPS compared with no recurrent UPS. PAWR expression in the recurrent group was lower than that in the no recurrent group (Fig. 4c). In the multivariable analysis, higher PAWR was an independent predictor of reduced recurrence risk (HR = 0.27, *P* = 0.0001; Table S12). However, survival analysis revealed no significant correlation between PAWR levels and clinical outcomes (HR = 0.96, *P* = 0.92; Table S13). PAWR (also named as Par-4) is a pro-apoptotic protein; its down-regulation is necessary and sufficient to promote recurrence in breast cancer [25]. Therefore, we next focused on PAWR for prediction of UPS recurrence. We examined PAWR expression in an independent validation cohort (*n* = 75) by immunohistochemical staining (Fig. 4d), and found that PAWR expression was negatively associated with UPS recurrence (Fig. 4e), indicating it as a protective factor. Our results suggest that PAWR is a negative predictive marker of UPS relapse.

### CD163 is a negative predictive marker for lung metastasis

**Fig. 5:**
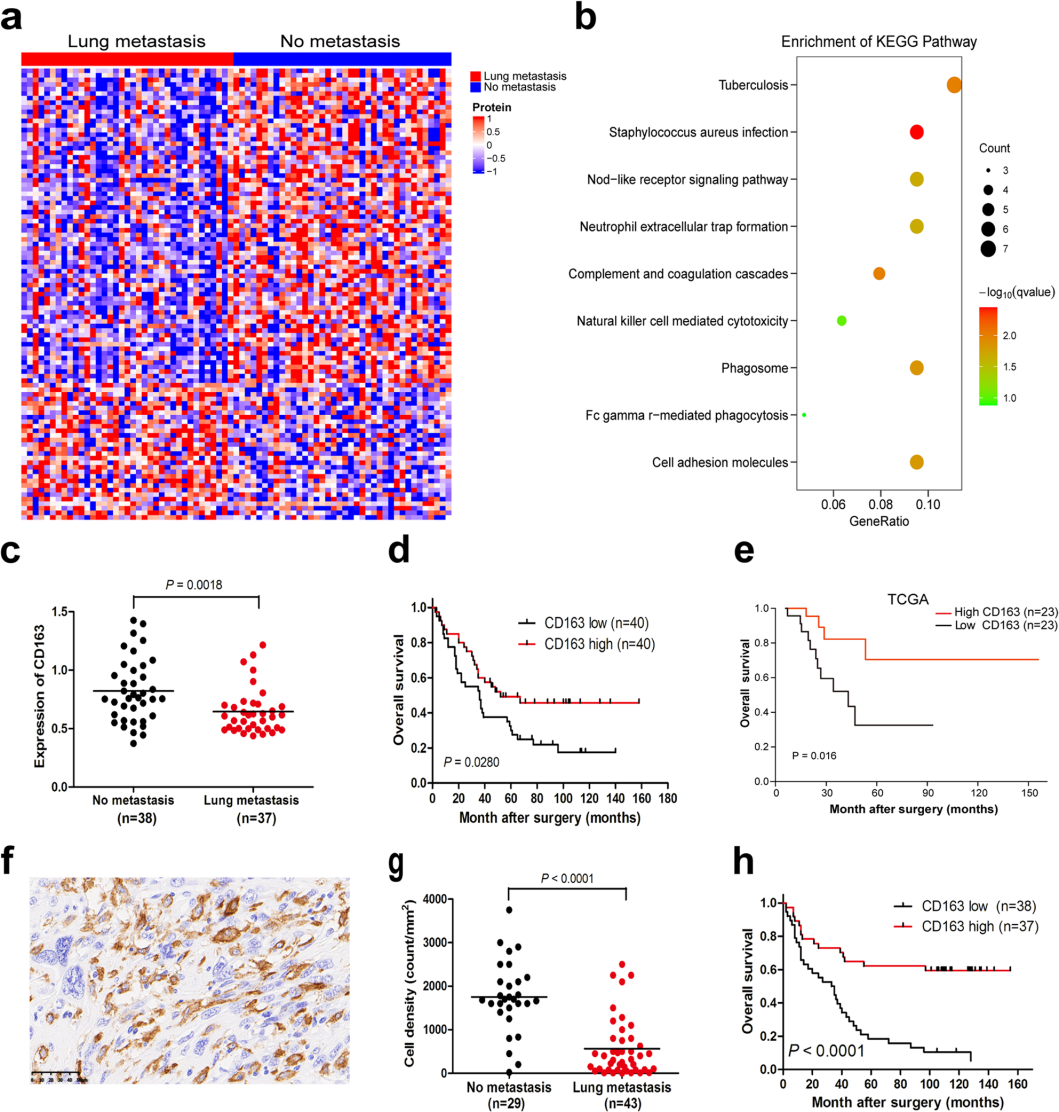
CD163 is a negative predictive marker for lung metastasis.** a** Consensus-clustering analysis of proteomic profiling in lung metastasis. **b** KEGG pathway enrichment analysis of lung metastasis. **c** Comparison of CD163 protein expression levels between no metastasis group and lung metastasis group in the proteomic cohort. **d** Kaplan-Meier curves of overall survival for CD163 expression levels in the proteomic cohort. **e** Kaplan-Meier curves of overall survival for CD163 expression levels in the TCGA UPS mRNA database. **f** Representative positive CD163 expression by immunohistochemical staining in UPS. **g** Comparison of CD163 protein expression levels between no metastasis group and lung metastasis group in the independent validation cohort. **h** Kaplan-Meier curves of overall survival for CD163 expression levels in the independent validation cohort

UPS is associated with a high rate (31% to 35%) of lung metastasis [1, 2]. Of the 80 patients in this study, 37 (46%) had lung metastasis in the long-term follow-up (44–158 months), 5 showed metastasis in other organs (bone and liver), and 38 had no metastasis. We next compared the proteomics profile between lung metastasis group and no metastasis group. Ninety-nine proteins were identified and significantly associated with lung metastasis (Table S14). Differential protein expression profiles were observed between the two groups (Fig. 5a). The KEGG pathway enrichment analysis showed that these proteins were focused on infection, phagosome, and cell adhesion molecules (Fig. 5b and Table S15), suggesting that immunity may play an important role in lung metastasis in UPS.

Interestingly, of the 99 proteins, CD163 was detected and considered as a macrophage marker. Macrophages are essential components of the immune microenvironment for UPS [7, 26], and CD163 + macrophages show phagocytic function [27, 28]. The CD163 protein level was significantly downregulated in UPS with lung metastasis compared with ones without lung metastasis (Fig. 5c; *P* = 0.0018). In the multivariable Cox regression for lung metastasis, CD163 was identified as an independent prognostic factor for reduced risk of lung metastasis (HR = 0.22, *P* = 0.04; Table S16). High CD163 expression was significantly associated with a favorable prognosis (Fig. 5d; *P* = 0.028) and also emerged as an independent favorable predictor in multivariable analysis (HR = 0.41, *P* = 0.01; Table S17). In accordance with our results, high CD163 expression was correlated with good clinical outcome in the TCGA UPS database (Fig. 5e; *P* = 0.016). To further confirm this, CD163 protein expression was detected in a new cohort (*n* = 75) by immunohistochemical staining (Fig. 5f). Of the 75 cases, three were excluded: two cases had only lymph node metastasis and one case had only bone metastasis. Results showed that CD163 was negatively associated with lung metastasis (Fig. 5g, *P* < 0.0001), and positively correlated with survival (Fig. 5h, *P* < 0.0001).

### Dissection of CD163 + macrophage by combining proteomics with single-cell RNA sequencing data

**Fig. 6:**
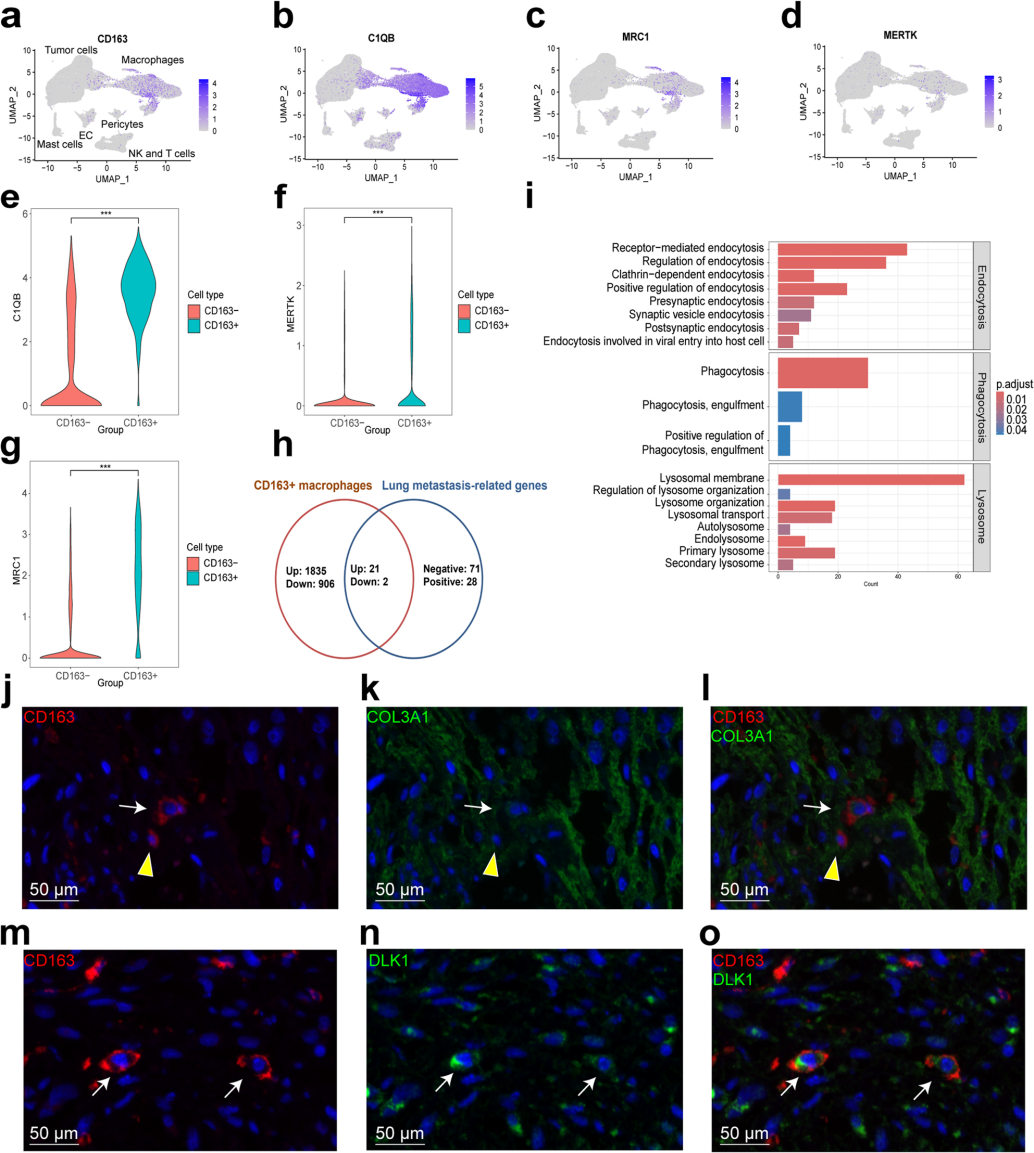
Dissection of CD163 + macrophage by proteomics and single-cell RNA sequencing. **a-d** Expressions of CD163, C1QB, MRC1, and MERTK in UPS subclusters at the single-cell resolution. EC: endothelial cells. **e-g** Expression of C1QB, MRC1, and MERTK between CD163 + macrophages and CD163- macrophages in UPS. **h** Screening of genes expressed in CD163 + macrophages from the 99 lung metastasis-related genes. **i** CD163 + macrophages were enriched in the endocytosis, phagocytosis, and lysosome compared with CD163- macrophages. **j-l** Phagocytosis of a COL3A1 (a fibroblast marker) positive tumor cell by a CD163 + macrophage (white arrow). Yellow arrow indicates a CD163 + macrophage which has a small nuclei compared with the tumor cell. **m-o** Co-expression of CD163 and DLK1 (a tumor subcluster marker) in UPS (white arrow)

Previously, we found that macrophages were the most abundant immune components in UPS using single-cell RNA sequencing [7]. However, the role of macrophages in UPS is largely unknown. In this study, CD163 was determined to be a negative predictor for lung metastasis in our two independent cohorts (Fig. 5c and g). We used our previous UPS single-cell RNA sequencing (scRNA-seq) data; CD163 was primarily expressed in macrophage cluster [7]. Macrophages are heterogeneous cell populations [27], including M1, M2, angiogenesis, and phagocytosis phenotypes; the signature markers for phagocytosis are CD163, MRC1, MERTK, and C1QB [27]. In UPS, expression of CD163, C1QB, MRC1, and MERTK are shown at the single-cell resolution (Fig. 6a–d) with MERTK showing low expression. We further analyzed the relationships between them in the UPS scRNA-seq data. CD163 was moderately correlated with MRC1 (*P* < 0.0001, *r* = 0.51), weakly correlated with MERTK (*P* < 0.0001, *r* = 0.37), and uncorrelated with C1QB (*P* = 0.02, *r* = 0.07), at single cell levels (Fig. S6). At protein levels, we did not detect MERTK by TMT-based proteomic analysis, which is in accordance with the MERTK result of scRNA-seq data (Fig. 6d). Interestingly, we found CD163 protein was highly correlated with MRC1 protein (*r* = 0.63, *P* < 0.0001), moderately correlated with C1QB protein (*r* = 0.40, *P* = 0.0002, Fig. S7). We further compared the gene expression between CD163 + macrophages and CD163- macrophages at single cell levels, the different expressed genes were shown in Table S18. Results showed that CD163 + macrophages had high expression of MRC1, MERTK, and C1QB (Fig. 6e–g). Interestingly, analyses of the pan-cancer myeloid cell scRNA-seq dataset [27] (*n* = 210 patients, Fig. S8) showed that MRC1, MERTK, and C1QB were also highly expressed in CD163 + macrophages compared with CD163- macrophages. PD-1/PD-L1 therapies function through a direct effect on macrophages; PD-1 expression on macrophages negatively correlates with phagocytic potency against tumor cells [29]. We found that PD-1 (PDCD1) expression was limited to T cells, and almost not expressed in macrophages and other cells in UPS (Fig. S9). These results indicate that CD163 + macrophages have high expression of phagocytosis-related genes and low expression of PD1.

To explore which of the 99 lung metastasis-related genes were associated with the CD163 + macrophages, we integrated different expressed genes in CD163 + macrophages (Table S18) with the 99 lung metastasis-related genes (Table S14). Interestingly, of the 99 lung metastasis-related genes, 21 were overexpressed, and two (FLNA, CNN2) were underexpressed in CD163 + macrophages compared with CD163-macrophages (Fig. 6h). Amongst these 23 genes, C1QC, LILRB4, SIGLEC1, SLC2A5, and VSIG4 were specifically expressed in the macrophage cluster at single cell levels (Fig. S10). Interestingly, SIGLEC1 (CD169) + and VSIG4 + macrophages have been reported to show protective functions in cancer metastasis [30, 31]. CYTH4, FCGR3A, GIMAP5, ITGAM, ITGB2, and PLEK were expressed in the macrophage cluster, and NK and T cell clusters (Fig. S11). The other genes (APOL1, ALDH1A1, CNN2, FLNA, IFI44L, IFIT1, LGALS3BP, MX1, SAMHD1, TBXAS1, and TNNT3) were expressed in tumor cluster and macrophages (Fig. S12). Our results indicate that the expression of the lung metastasis-related genes is limited to particular cell clusters at single cell levels, in UPS.

Interestingly, chemokines (CCL14, CCL13, CCL18, CCL8, and CXCL12) were significantly upregulated in CD163 + macrophages compared with CD163- macrophages (Fig. S13 and Table S18). In addition, phagocytosis-associated molecular CD209 and co-stimulation factor CD28 showed abundance in CD163 + macrophages compared with those in CD163- macrophages (Fig. S14 and Table S18). In addition, GO functional enrichment analysis showed that CD163 + macrophages were enriched in endocytosis, phagocytosis, and lysosome compared with CD163- macrophages (Fig. 6i). Furthermore, IF confirmed the co-expression of CD163 and COL3A1 (a fibroblast marker) or DLK1 (a tumor subcluster marker) in UPS (Fig. 6j–o). The phagocytized cells with positive CD163 expression had larger nuclei than macrophages, and expressed the fibroblast marker COL3A1 or tumor subcluster marker DLK1, which was within the location of CD163, indicating phagocytosis in UPS, mediated by CD163 + macrophages.

Tumors are complex ecosystems comprised of diverse cell types that interact with each other. Therefore, we dissected the interaction network between CD163 + macrophages and other cells (Table S19). As expected, CSF1 − CSF1R interaction were observed (Fig. S15), which controls the production, differentiation, and function of macrophages. TNF, TGF-β, and chemokine signaling were abundant (Fig. S15). Interestingly, we found that other cells acted through the PTPRC−MRC1, CEACAM1 − CD209, ICAM3 − CD209, and ICAM2 − CD209 interaction on the CD163 + macrophage to promote phagocytosis [32]. In addition, interactions (CD80 − CD28, CD86 − CD28, CD86 − CTLA4, SIRPG−CD47, SIRPA−CD47, CD40LG−CD40) between CD163 + macrophage and other cells were observed (Fig. S15), which were vital regulators of immune signal transduction. Based on the fact that macrophages are the most abundant immune cells in UPS, our results highlight the potential cellular interactions that shape the UPS microenvironment.

## Discussion

The pan-STS proteomic studies have broadened our understanding of the heterogeneity in various sarcomas [13, 14]. However, detailed proteomes of several sarcoma subtypes in a large cohort remain largely unexplored. Herein, our global proteomic analysis provided new and deep insights into UPS biology. To our knowledge, this is the largest cohort (*n* = 80) study of UPS proteomics. Previously unknown molecular-based classification, candidate therapeutic targets, local relapse and lung metastasis-related markers were revealed, thus highlighting the importance of proteomic analysis in better understanding malignant UPS.

The current TNM stage classification [33] for STS is dependent on the histologic grade (G), tumor size (T), lymph node metastasis (N) and distant metastasis (M), which shows limited clinical values in practice. For example, patients with UPS or clear cell sarcoma or synovial sarcoma, having the same T, N, and M, may be predicted to have similar prognosis based on the TNM stage classification; however, they have different genetic backgrounds, pathologic characteristics, and responses to chemotherapy and immunotherapy; besides, there are no stage I patients, as the G is 2 at least, for the high-graded sarcoma types. In accordance with this, patients with UPS were not well classified by the recommended AJCC staging system (Fig. 2c). In contrast, protein-based disease classification reliably stratified patients into different prognostic groups (Fig. 2d). Our results highlight the utility of a biological signature approach based on proteomics, in refining clinical risk stratification for STS.

UPS is a heterogeneous disease, and has no proteomic-based molecular subtype to guide clinical treatment [13, 14, 34]. In this study, we divided UPS patients into three subtypes based on UPS proteomics, a high-risk subtype S-I, immune subtype S-II, and a low-risk subtype S-III, which may provide guidance for clinical treatment. Subtype S-I was characterized by the high expression of spliceosome-related proteins and proliferative protein MKI-67 (Fig. 2a and b); overexpression of the spliceosome-related proteins may lead to the misregulation of alternative splicing and eventually contribute to the worst prognosis. Interestingly, PRMT6 was highly expressed in S-I (Fig. S4). PRMT6, an integral methyltransferase, participates in regulating DNA base excision repair [35]. PRMT6 is frequently overexpressed in human breast cancer [23], glioblastoma [19], and colon tumor [36], and its expression contributes to tumor malignancy, recommending PRMT6 as a potential target. In this study, we found that PRMT6 protein was overexpressed by almost 4-fold in UPS compared with that in their corresponding nontumorous tissues (median = 0.82 and 0.21, respectively; Fig. 3b), elevated PRMT6 expression was correlated with poor prognosis (Fig. 3a and f), and PRMT6 inhibitor (EPZ020411) significantly inhibited tumor growth in two UPS PDX models (Fig. 3h and k). Our results underscore the prospective utility of PRMT6 inhibitor as a therapeutic strategy against UPS in patients with the subtype S-I. The greatest response to anti-PD1 therapy was observed in patients with UPS subtype of advanced STS [37]. However, the heterogeneity of the immune microenvironment within UPS makes it a challenge to distinguish which patients respond to immune therapy. Interestingly, we defined a subtype (S-II) of UPS which was characterized by enriched monocytes, macrophages, MHC I, MHC II, and the highest ImmuneScore (Fig. 2a and b, and Fig. S2). Sarcoma ecotypes with macrophage infiltration predict response to immunotherapy [38]; thus, patients with UPS with subtype S-II may benefit more from immunotherapy. UPS subtype S-III showed the largest amounts of endothelial cells (Fig. S2), which supports the use of anti-VEGFR therapy (pazopanib and anlotinib) for this subtype. In addition, integrating proteomics with the translational strength of UPS patient-derived primary cultures [39, 40] may represent a highly promising strategy to guide patient treatment.

UPS has a high local recurrence rate of 19–31% [1, 2]. However, the causes and mechanisms underlying this recurrence remain unclear, with scant research in this area. Our proteomic analysis identified an association between PAWR (also known as Par-4) and UPS recurrence, which was further confirmed in an independent validation cohort (*n* = 75) using immunohistochemical staining (Fig. 4d and e). This suggests that evaluating PAWR protein levels could be utilized to assess the risk of UPS recurrence. Among the 46 UPS cases in the TCGA database, recurrence information was not available. Therefore, we could not assess the correlation between PAWR expression and recurrence in these 46 patients. Consistent with our results, PAWR is a known tumor suppressor; its downregulation or loss is a key driver of recurrence in breast cancer, and restoring its expression in recurrent tumor cells can lead to multinucleation, p53 activation, growth arrest, and apoptosis [25]. In addition to apoptosis, PAWR plays a significant role in regulating ferroptosis, a process mechanistically driven by the autophagic machinery [41]. Zhao et al. demonstrate that the nSMase2/MAPK/NF-κB pathway mediates the tumor-inhibitory effect induced by PAWR activation in esophageal squamous cell carcinoma [42]. In our study, PAWR was the most downregulated protein in recurrent UPS compared with non-recurrent cases. Therefore, restoring PAWR expression may represent a promising strategy to prevent UPS relapse.

In STS, the role of CD163 + macrophages for prognosis is controversial. High levels of CD163 + macrophages are recognized as a poor prognostic factor in synovial sarcoma, myxoid liposarcoma [43], whereas it is a positive prognostic indicator for dedifferentiated liposarcoma [26], ewing sarcoma, and osteosarcoma [43]; these are likely related to macrophage plasticity and resultant heterogeneity of phenotype in different immune microenvironments. In UPS, high CD163 + macrophage infiltration was associated with a good overall survival in primary UPS (*n* = 46) [44], which is consistent with the data from the TCGA UPS cohort and our two independent cohorts (Fig. 5d and e, and 5h). UPS shows high rates of lung metastasis [1, 2]. However, no immune cells are reported to be associated with lung metastasis. To our knowledge, we are the first to discover that CD163 + macrophages are negative predictive markers of lung metastasis in UPS. Therefore, patients with low levels of CD163 + macrophages should be monitored closely and undergo aggressive treatment for UPS.

Macrophages are essential members of the innate immune response. They have important functions including phagocytosis, angiogenesis regulation, and immunomodulation [27]. Macrophages are typically divided into M1 (anti-tumor) and M2 (pro-tumor) types [43]. Macrophages are heterogeneous, exhibiting more complex phenotypes, which argues against such a simple categorization. At the single cell level, macrophages can be classified as M1, M2, angiogenesis, and phagocytosis phenotypes [27]. In this study, the CD163 + macrophages were characterized by high expression of MRC1, C1QB, CCL14, CCL13, CCL18, CCL8, CXCL12, CD209, and CD28, and low expression of PD1. GO functional enrichment analysis showed that CD163 + macrophages were enriched in the endocytosis, phagocytosis, and lysosome. Phagocytosis of tumor cells by CD163 + macrophages was observed in UPS (Fig. 6j–o). Currently, CD163 is frequently considered as an M2 type marker, promoting tumor progression [43]. However, based on our results, it is more appropriate to classify CD163 + macrophages in UPS, as an independent subtype with phagocytosis. CD163 + macrophages interacted with other immune cells to shape the UPS microenvironment (Fig. S15). Therefore, the presence of CD163 + macrophages is indicative of not only specific phagocytic activity but also a broader inflammatory or immune-active state [45], collectively pointing to their role as key modulators of the tumor immune landscape. Interestingly, macrophages in sarcoma can determine response to immune checkpoint inhibition [38, 46], activate the adaptive immune system, and directly kill tumor cells in response to immune checkpoint inhibitors [29]. UPS and dedifferentiated liposarcoma show better response to immunotherapy than other sarcoma types, which may be explained by the observation that UPS and dedifferentiated liposarcoma have relatively high counts of macrophages among different histological types of sarcomas [26]. Our results highlight the roles of CD163 + macrophages in the UPS immune microenvironment, which may have implications for research on macrophages in other sarcoma types and solid tumors.

## Conclusions

Our study identifies the proteomic landscape of UPS, and the proteomic subtypes of UPS correlates with different clinical outcomes. PRMT6 inhibitor may be a novel promising approach for treating UPS subtype S-I; advanced immunotherapy may be considered in the treatment of subtype S-II, while anti-angiogenesis treatments may be recommended for subtype S-III. In addition, PAWR and CD163 + macrophages can be used as predictive markers of local recurrence and lung metastasis, respectively. Our study not only provides biological insights underlying clinical features of UPS but also propels us towards the era of proteomics-driven precision medicine in sarcoma research.

## Supplementary Information

Supplementary Material 1.

Supplementary Material 2.

## Data Availability

The raw ﬁles of proteome dataset can be obtained from iProX database (https://www.iprox.cn/page/MSV022.html, accession number IPX0012435000). Single-cell sequencing data of human UPS specimens has been deposited at Genome Sequence Archive (GSA) with the identiﬁer: HRA004389. TCGA SARC RNA-seq and survival data were downloaded from the Genomic Data Commons (GDC) (https://portal.gdc.cancer.gov/).

## Abbreviations

ATCC: American Type Culture Collection
AJCC: American Joint Committee on Cancer
ECM: Etracellular matrix
GO: Gene Ontology
GSEA: Gene Set Enrichment Analysis
HR: Hazard ratio
IF: Immunofluorescence
IHC: Immunohistochemistry
KEGG: Kyoto Encyclopedia of Genes and Genomes
LC-MS: Liquid Chromatography-Mass Spectrometry
MFH: Mlignant fibrous histiocytoma
PCA: Principal component analysis
PDX: Patient-derived xenograft
scRNA-seq: Single-cell RNA sequencing
STS: Soft tissue sarcomas
TCGA: The Cancer Genome Atlas
TME: Tumor microenvironment
TMT: Tandem mass tag
TNM: Tumor-Node-Metastasis
UPS: Undifferentiated pleomorphic sarcoma
