# Supplementary material for: Proteomic landscape analysis of undifferentiated pleomorphic sarcoma
Source: Genome Med. 2026 Mar 20;18:50. doi: 10.1186/s13073-026-01626-w (PMC13127049; doi:10.1186/s13073-026-01626-w)
Supplement: Supplementary file 1 — Supplementary Material 1. [file 13073_2026_1626_MOESM1_ESM.docx]

**Supplementary figures：**


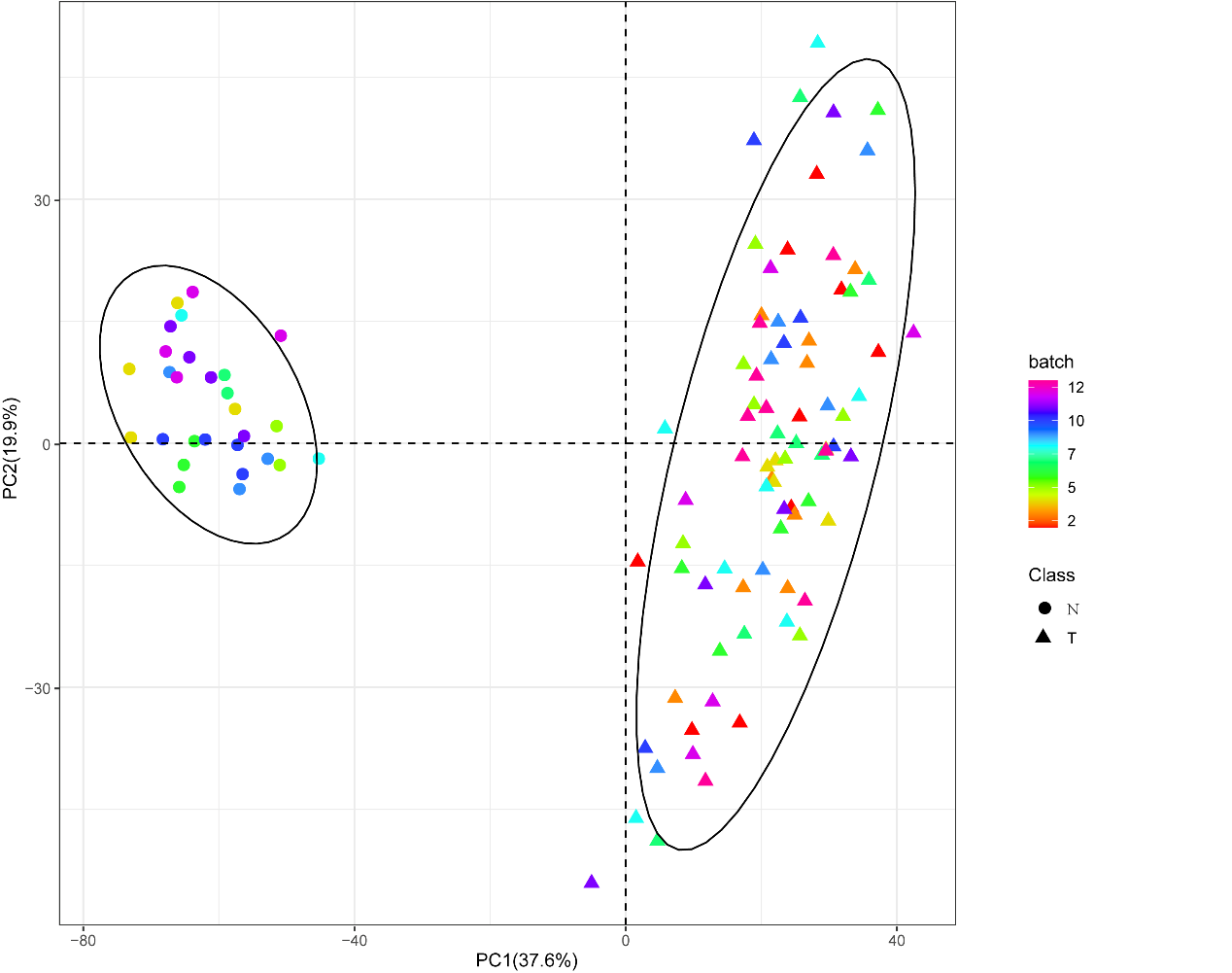


**Fig. S1.** Principle component analysis (PCA) of the proteomic data separated tumor samples (T) from non-tumor (N) samples, and no batch effects were observed. Samples analyzed in different groups (batches) are shown with different colors.


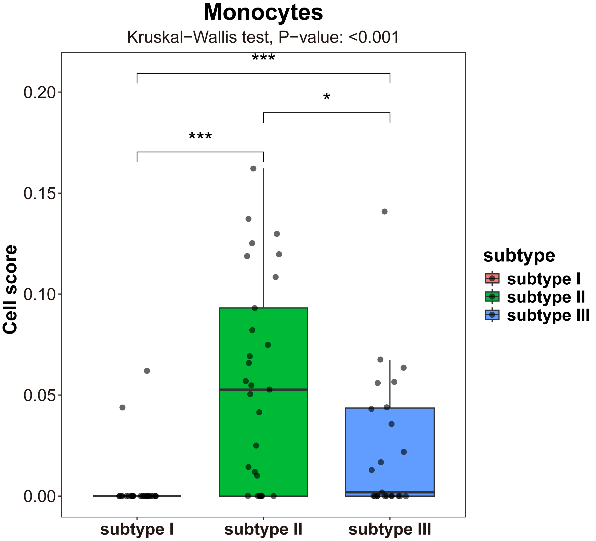

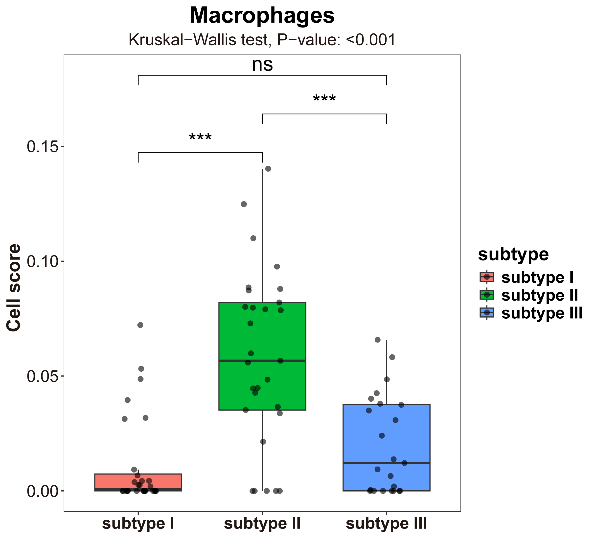


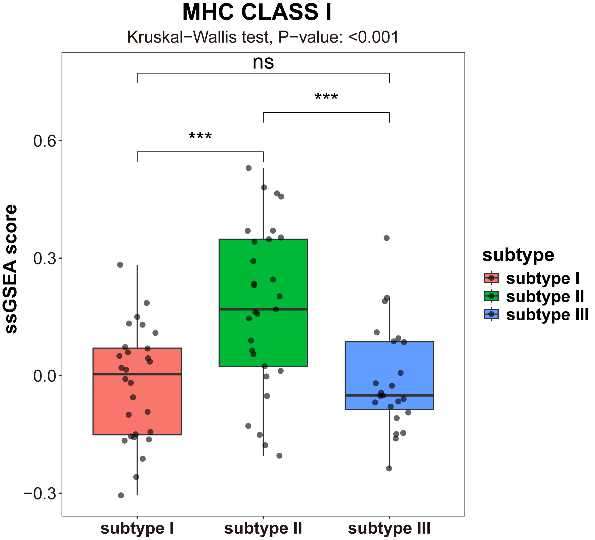

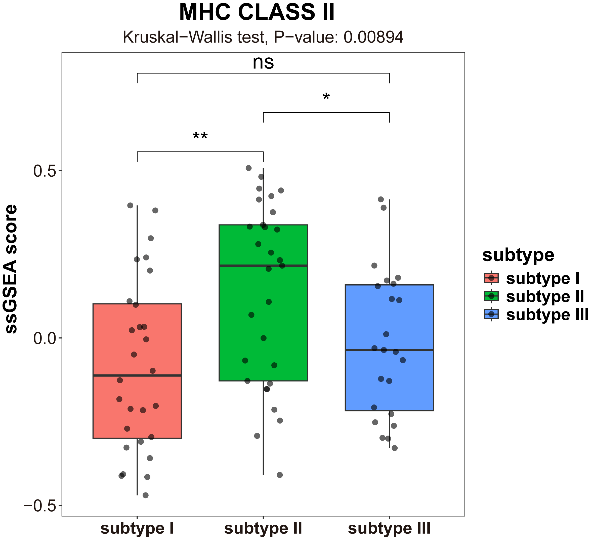


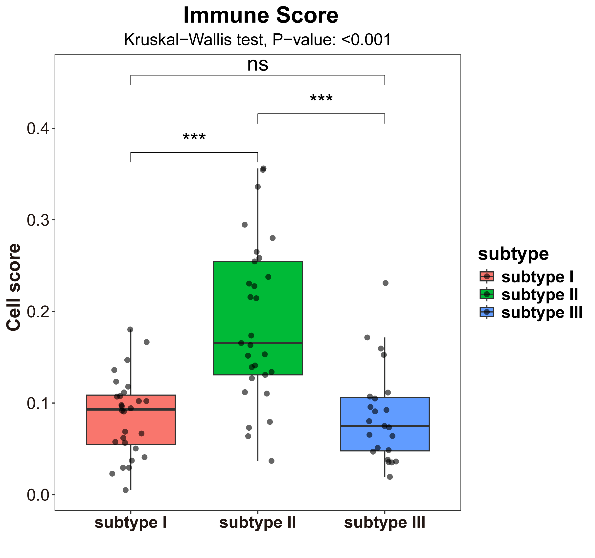

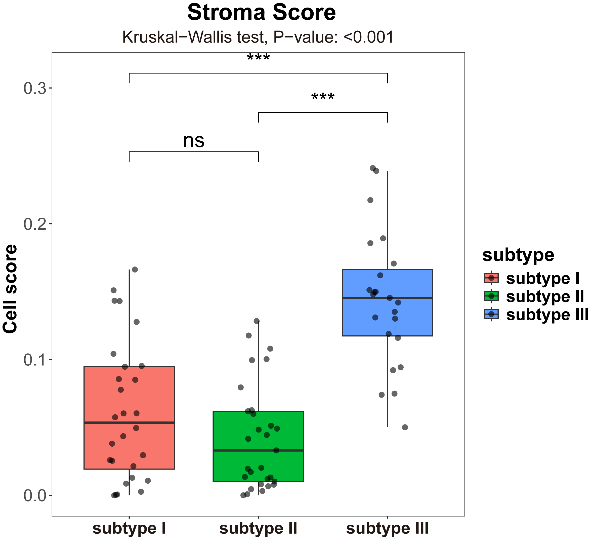


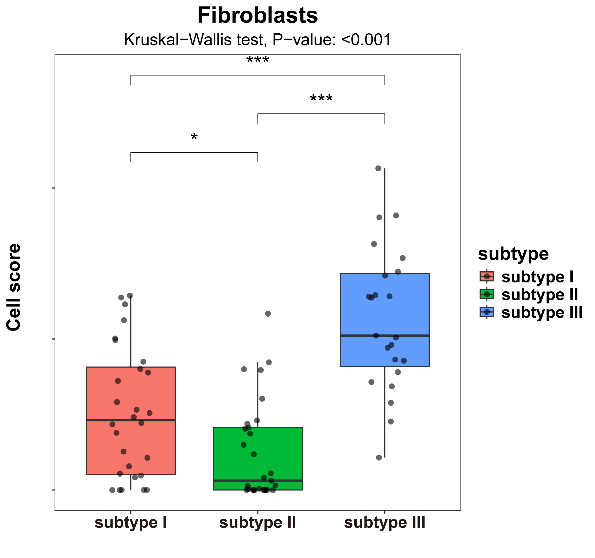

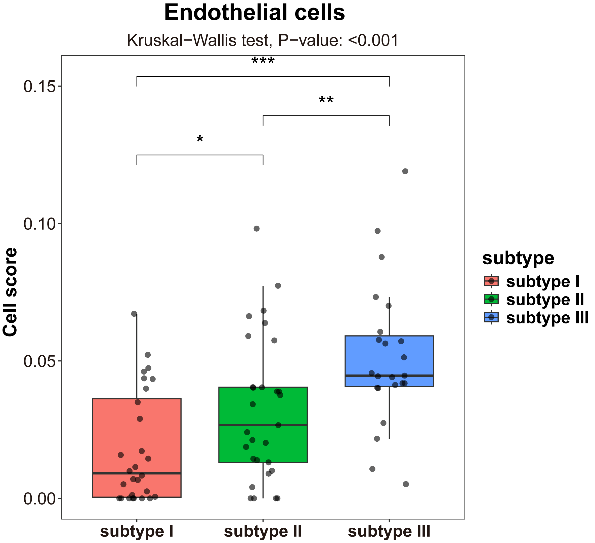


**Fig. S2.** Analysis of cell scores for monocytes, macrophages, fibroblasts and endothelial cells, and MHC I, MHC II, ImmuneScore, and StromaScore in the three proteomic subtypes.


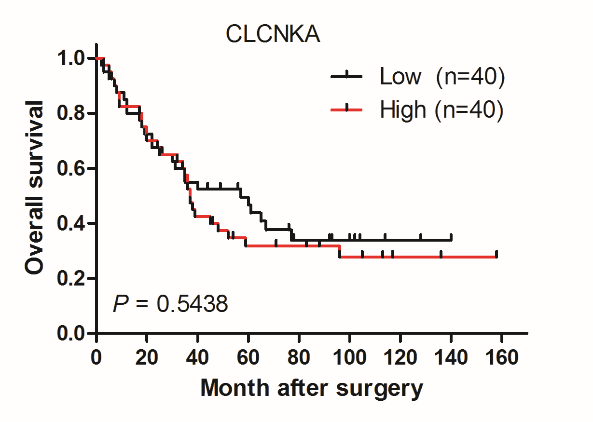

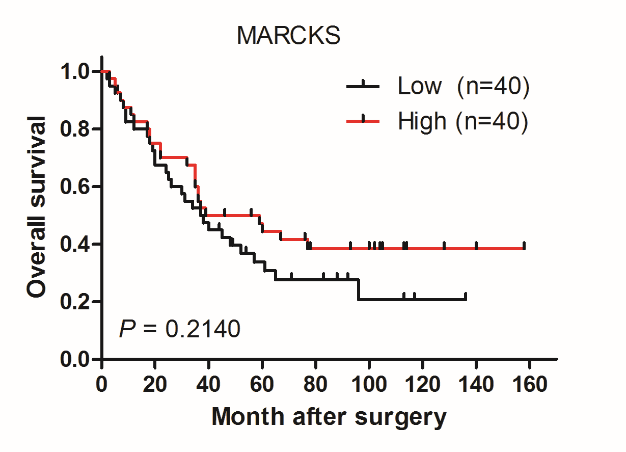


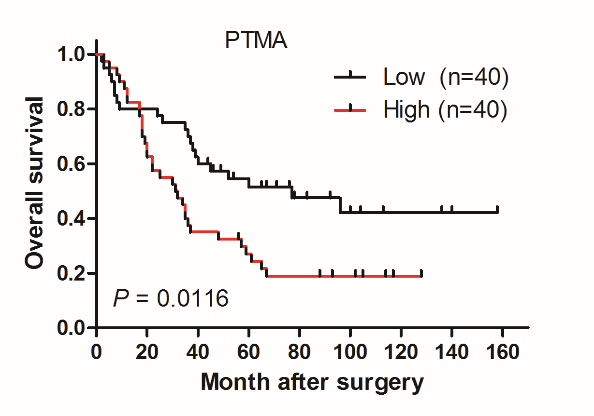

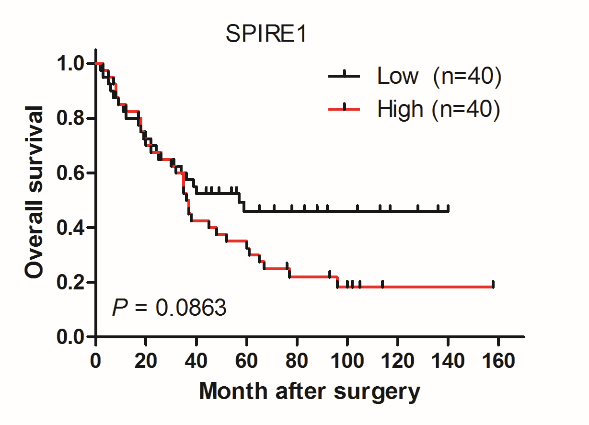


**Fig. S3.** Association of CLCNKA, MARCKS, PTMA, and SPIRE1 with prognosis, in 80 patients with UPS.


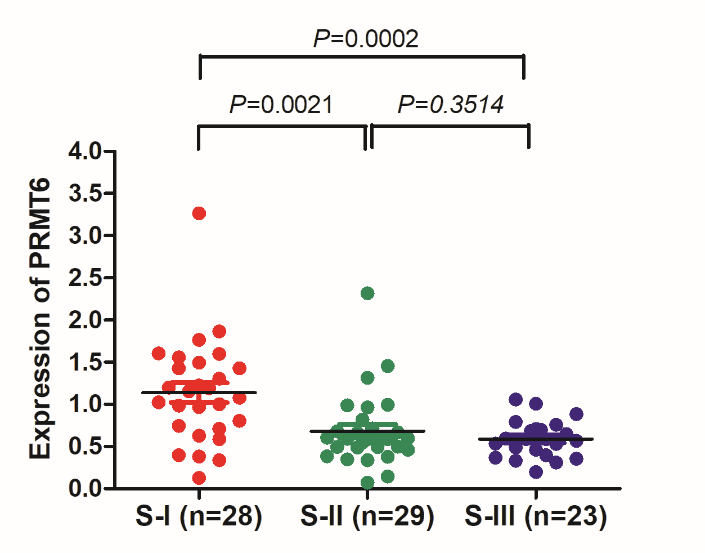


**Fig. S4.** PRMT6 was highly expressed in S-I.


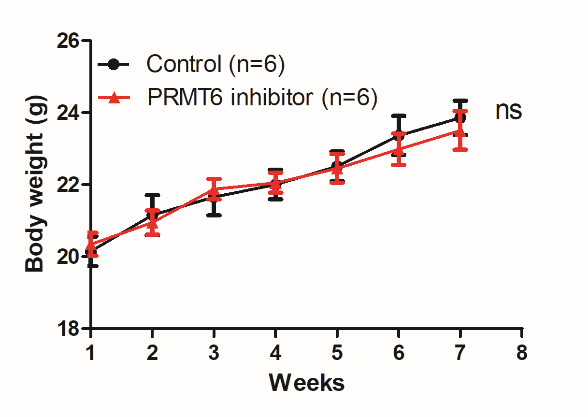

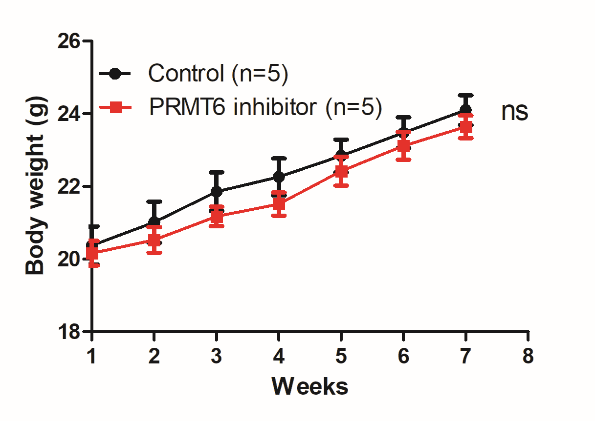


**Fig. S5.** EPZ020411 had no effect on body weight.


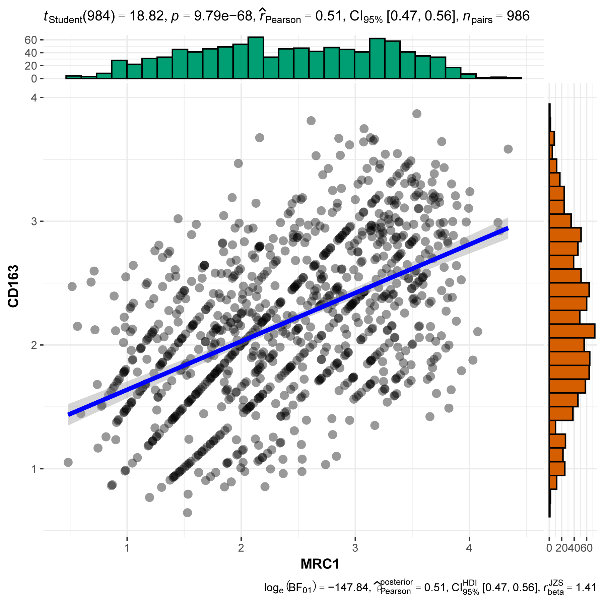


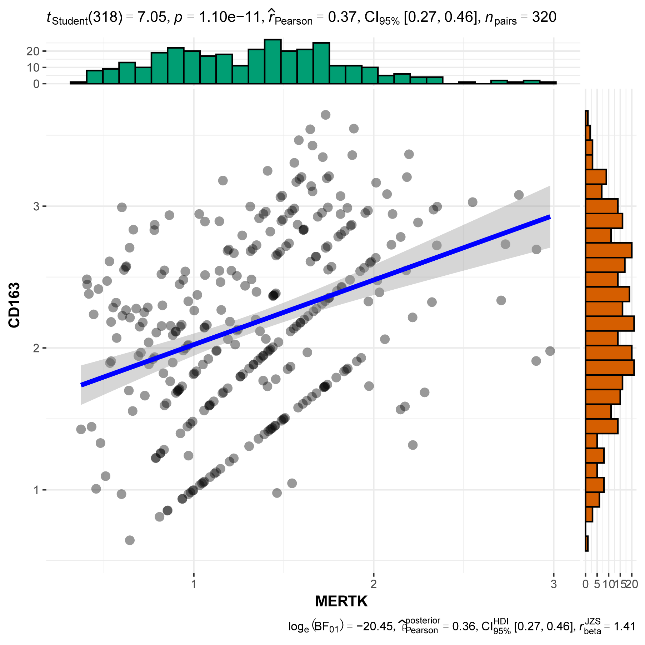


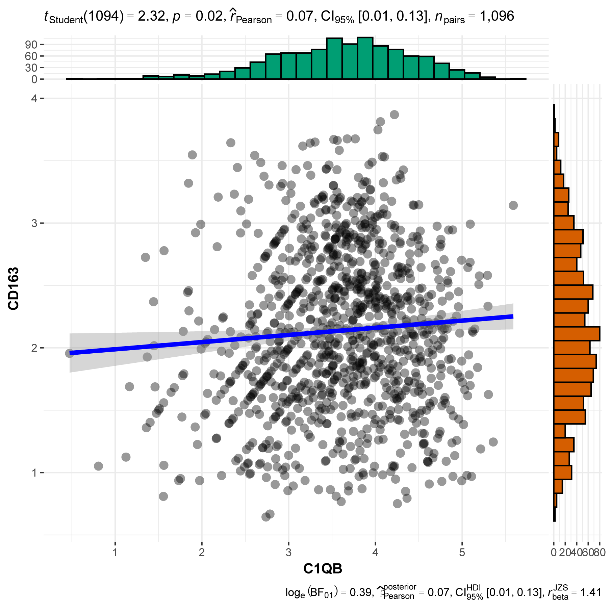


**Fig. S6.** The relationships of CD163 with MRC1, MERTK, and C1QB at single cell levels.


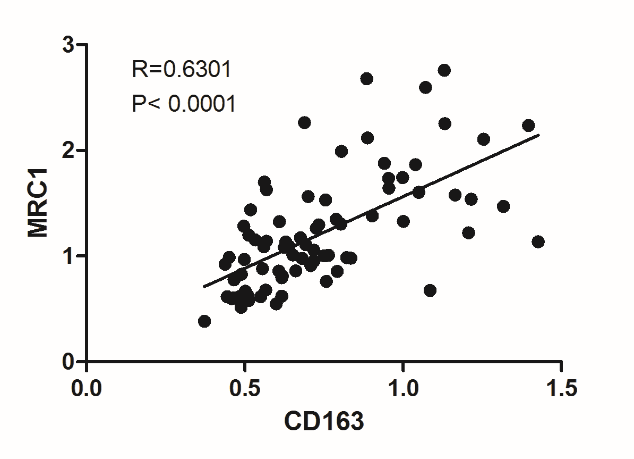

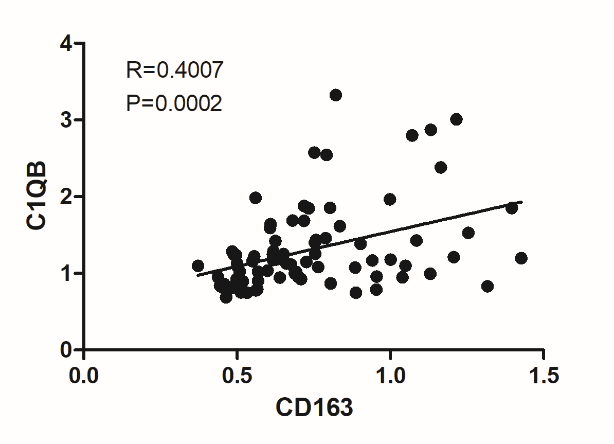


**Fig. S7.** CD163 protein was correlated with MRC1 and C1QB proteins in 80 patients with UPS.


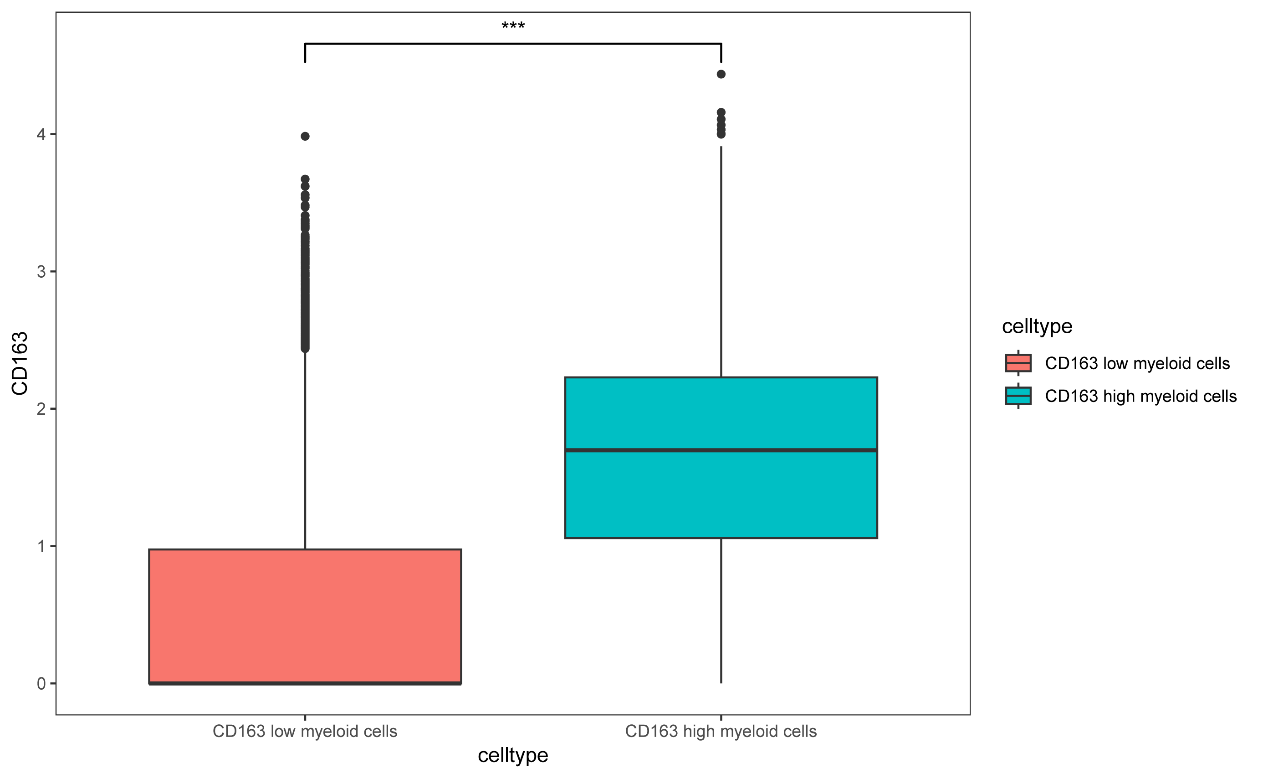


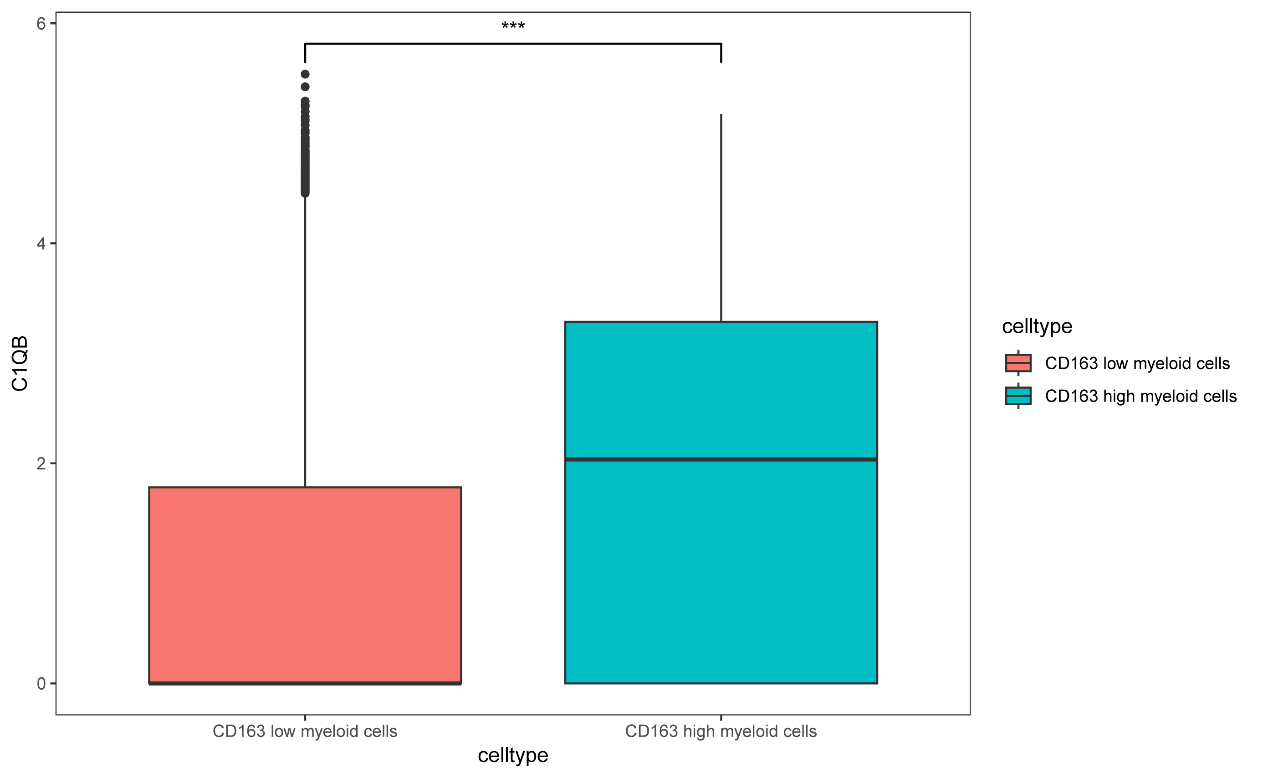


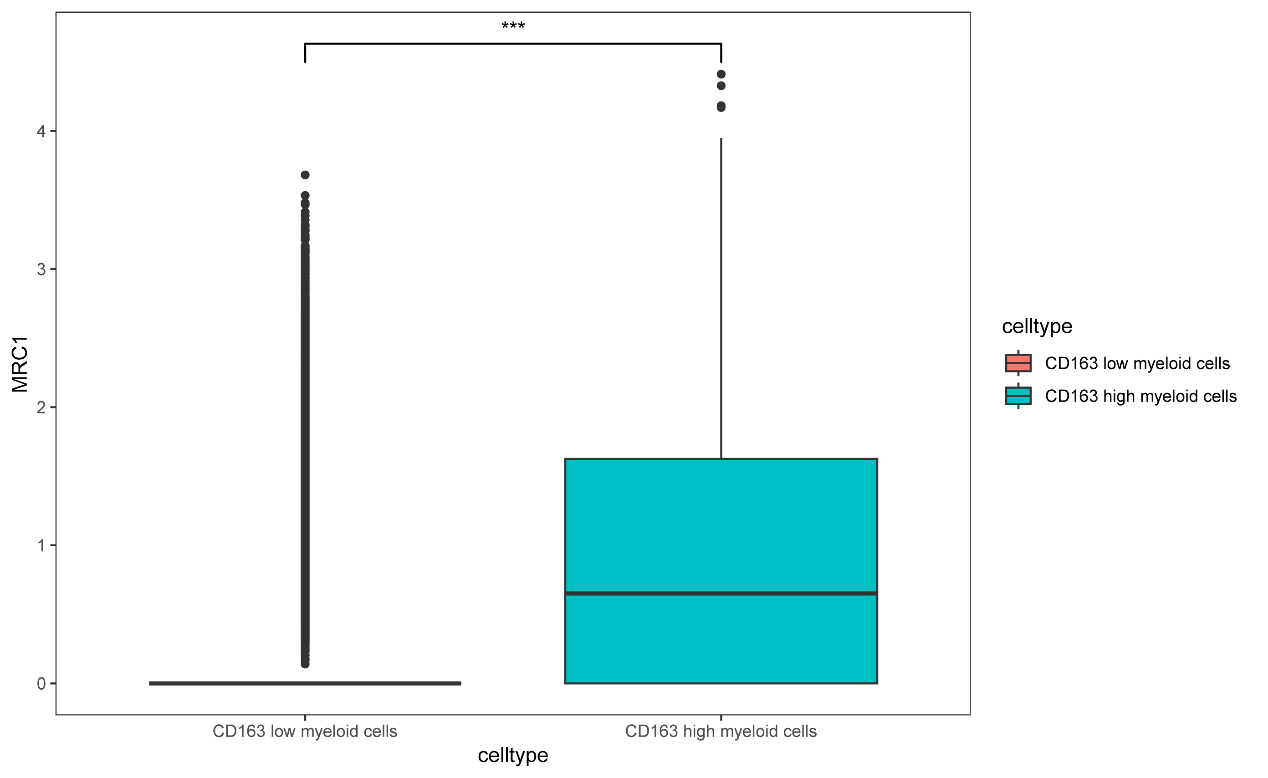


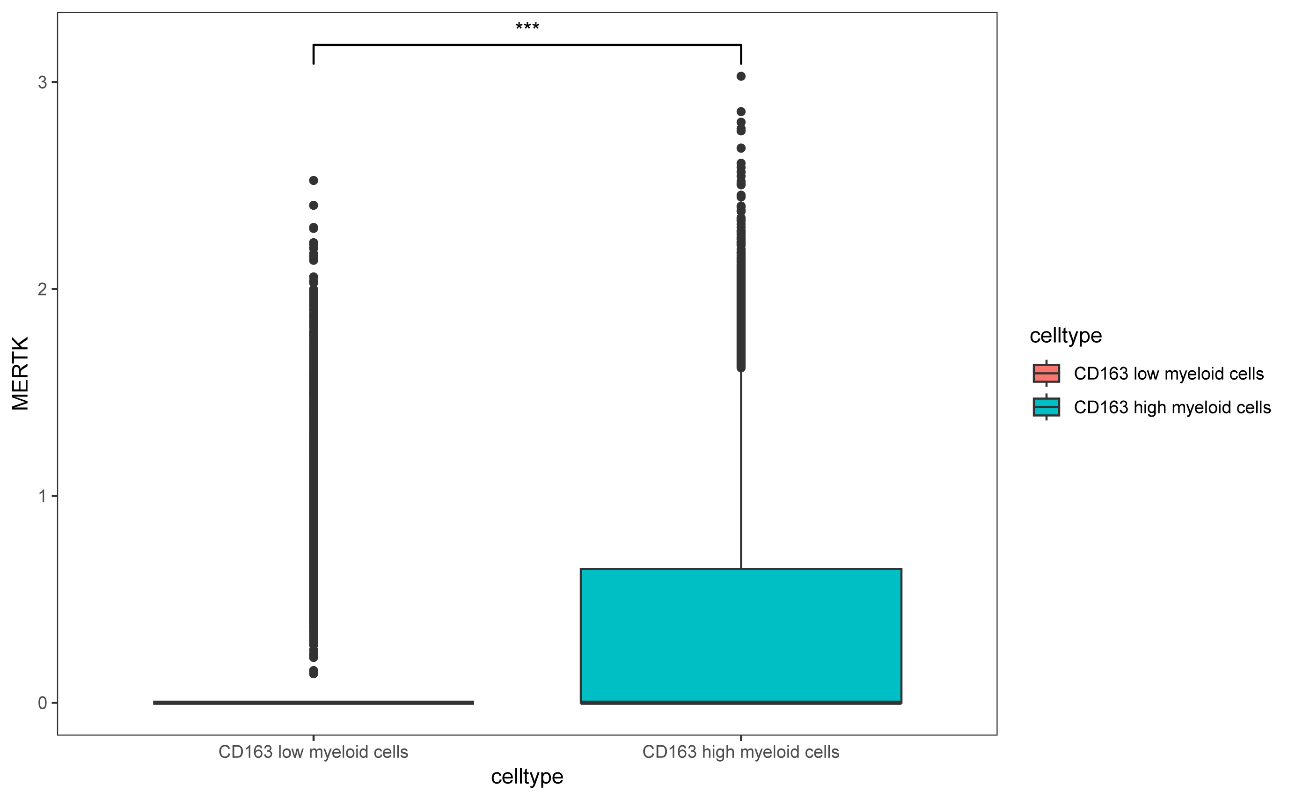


**Fig. S8.** The expression of CD163, C1QB, MRC1, and MERTK in CD163+(high) macrophages compared with that in CD163-(low) macrophages, by analyzing the pan-cancer myeloid cell scRNA-seq dataset (210 patients).


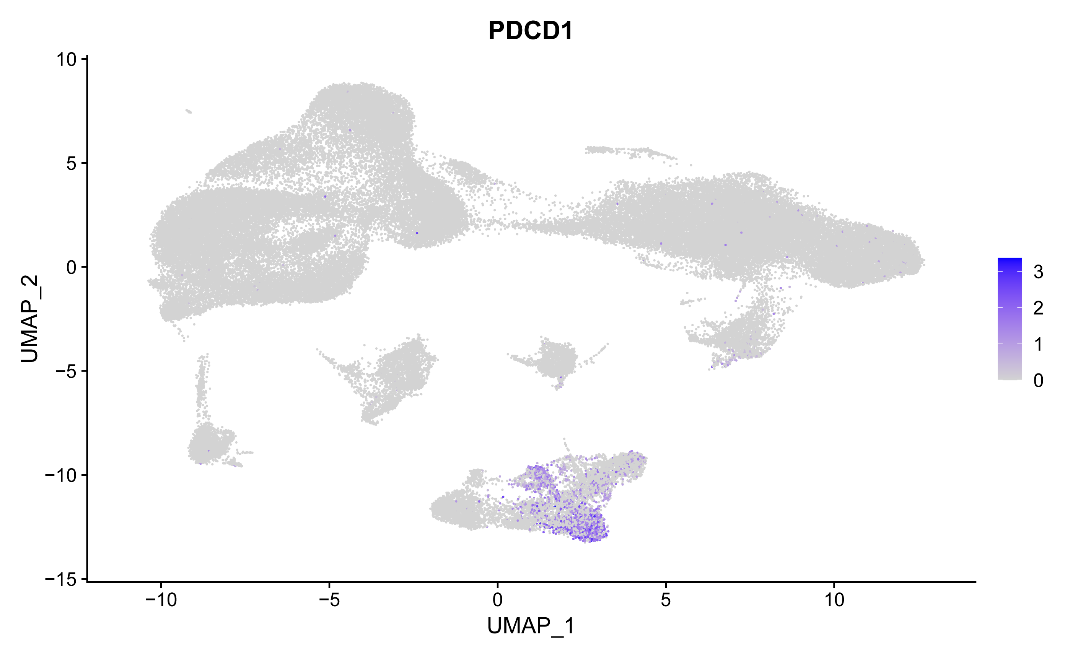


**Fig. S9.** PDCD1 (PD-1) expression in UPS, is limited to T cells.


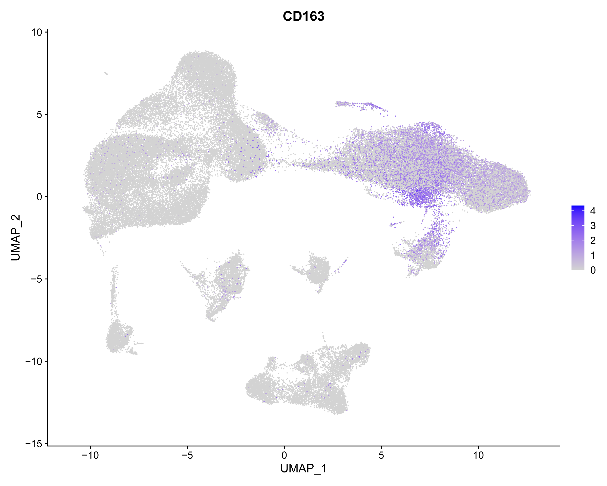

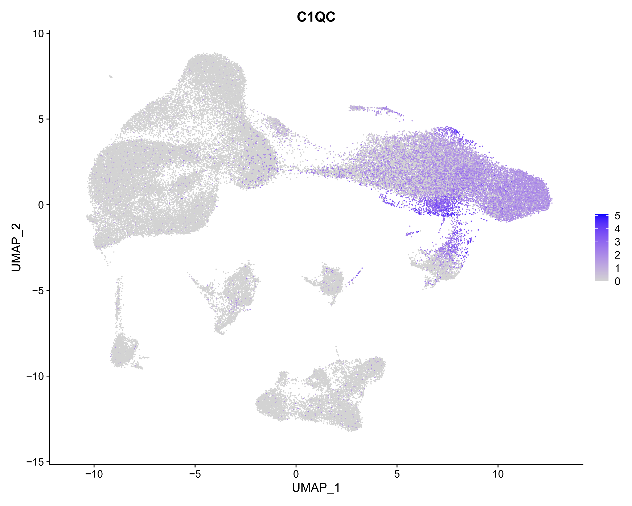


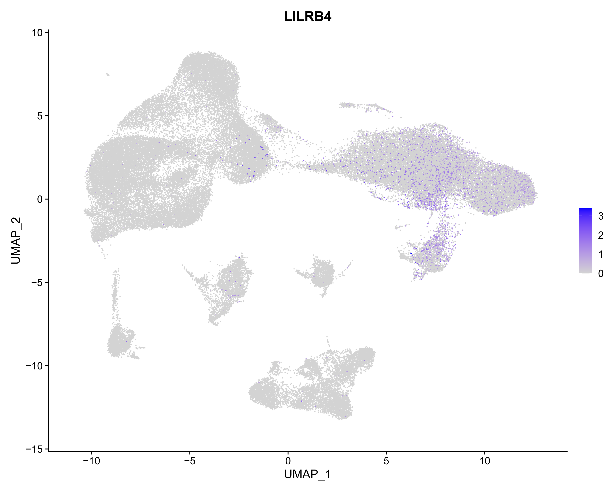

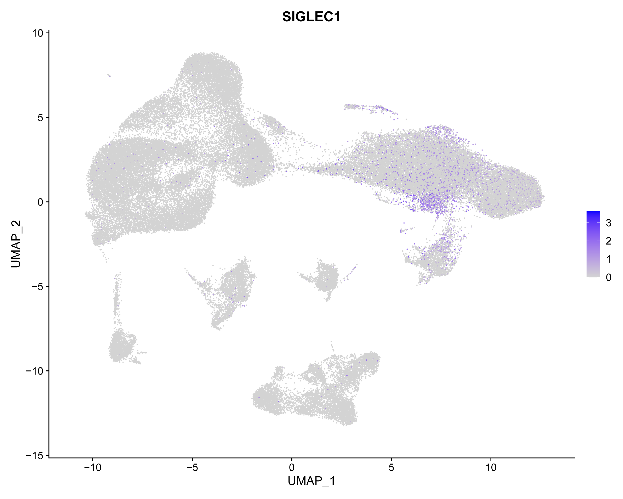


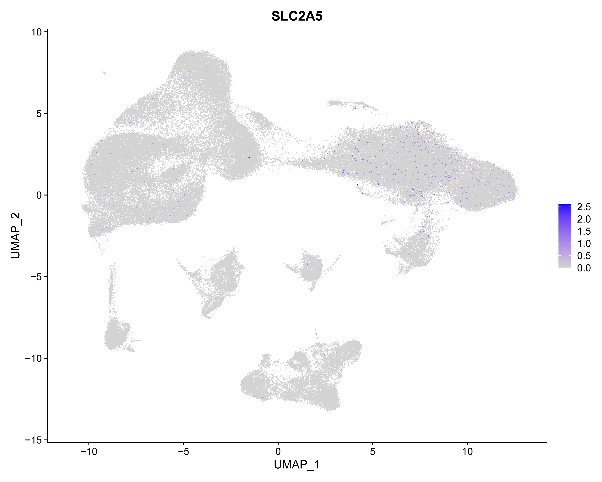

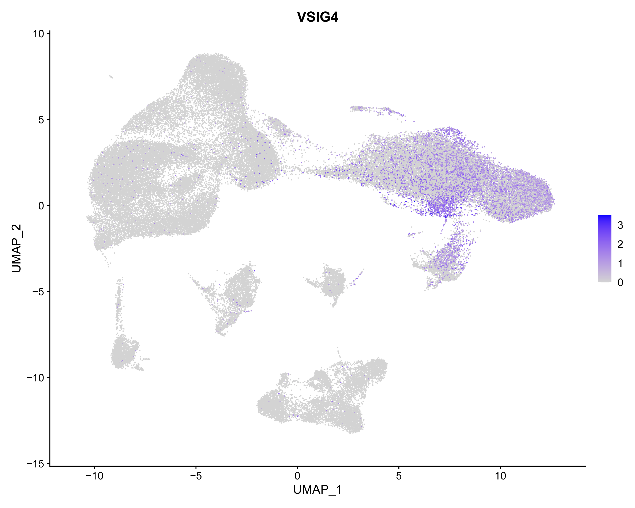


**Fig. S10.** CD163, C1QC, LILRB4, SIGLEC1, SLC2A5, and VSIG4 were specifically expressed in the macrophage cluster.


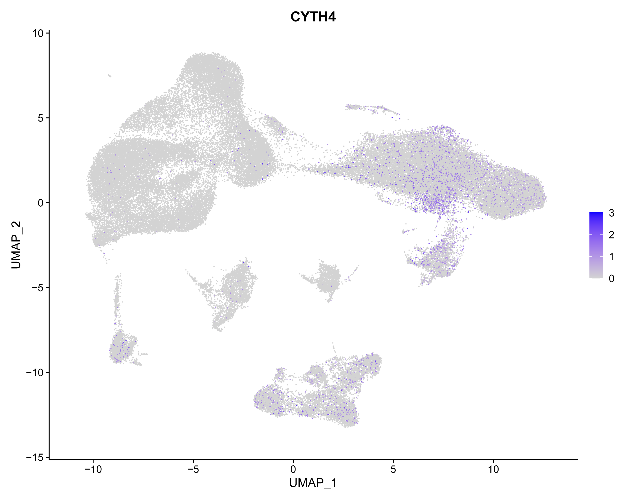

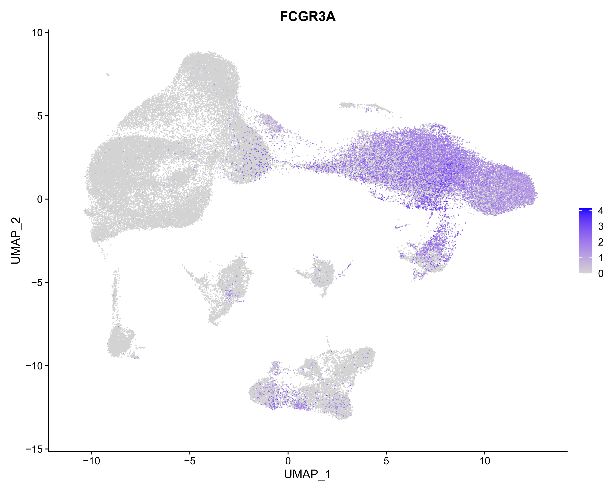


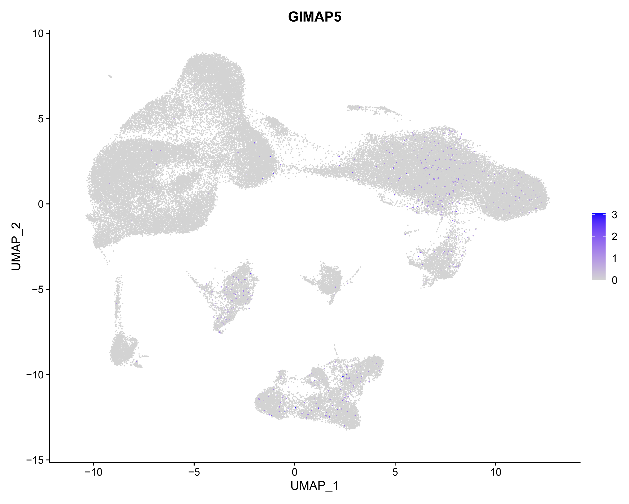

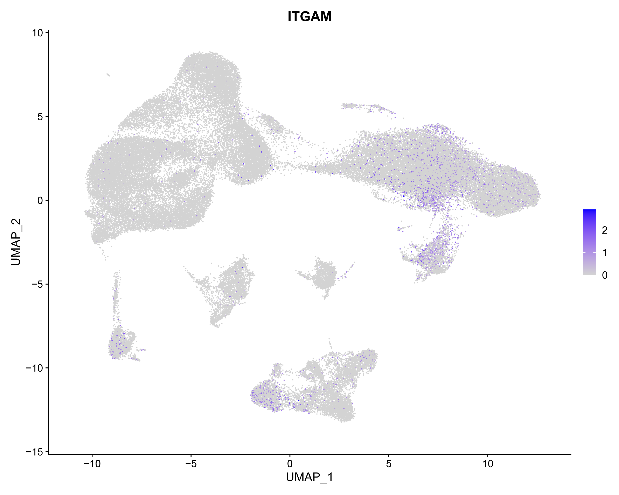


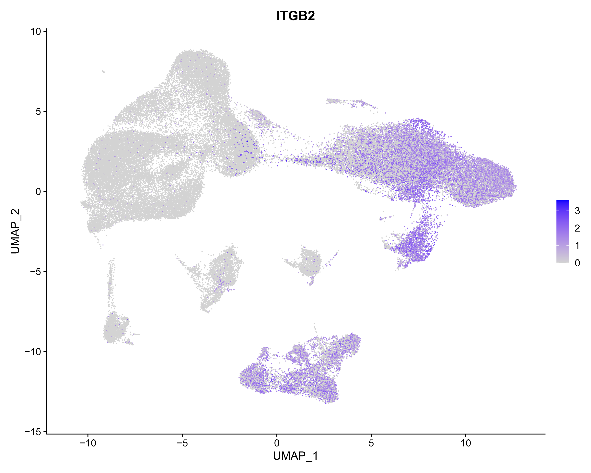

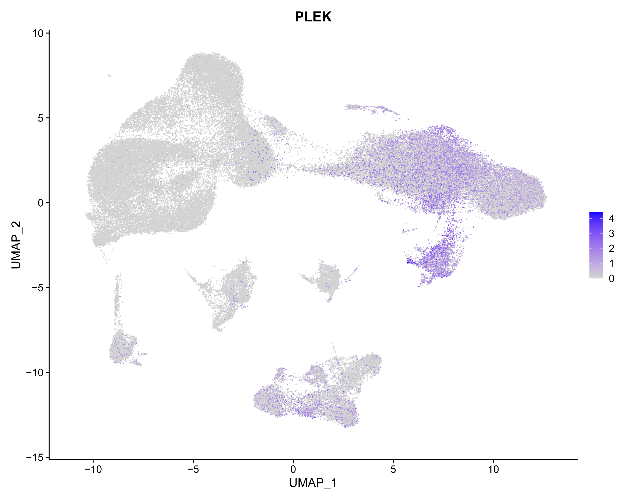


**Fig. S11.** CYTH4, FCGR3A, GIMAP5, ITGAM, ITGB2, and PLEK were expressed in the macrophage cluster, and NK and T cell clusters.


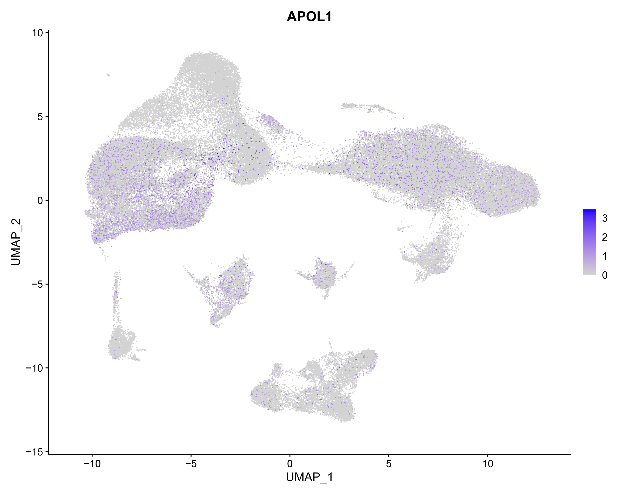

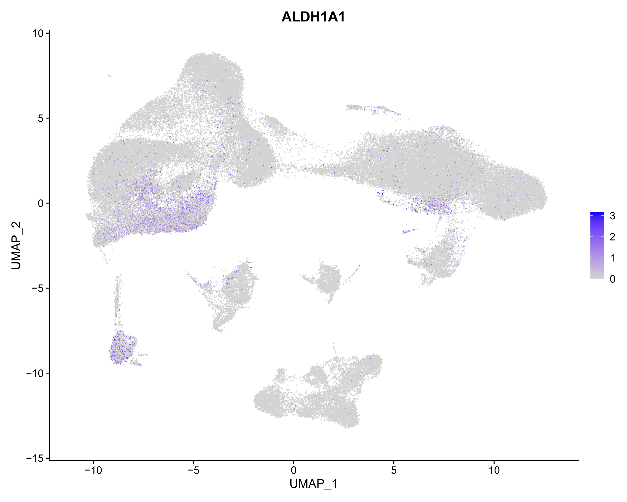


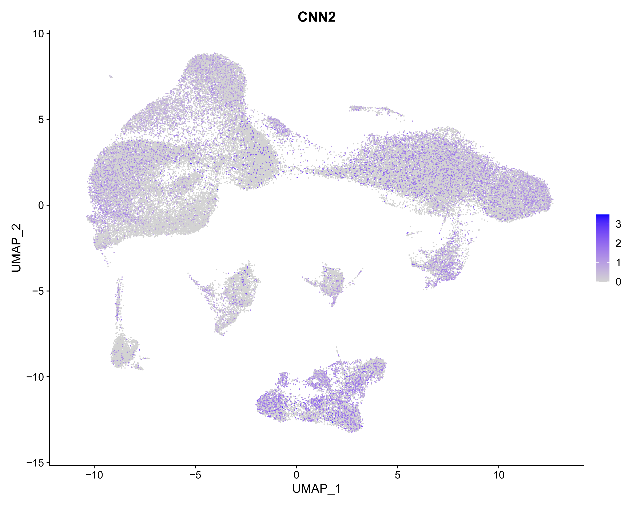

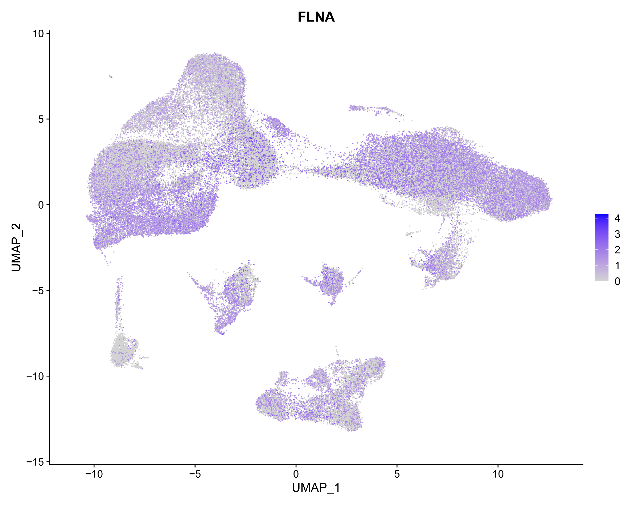


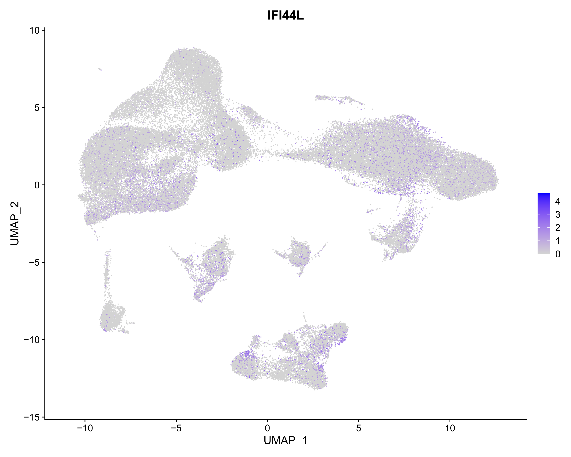

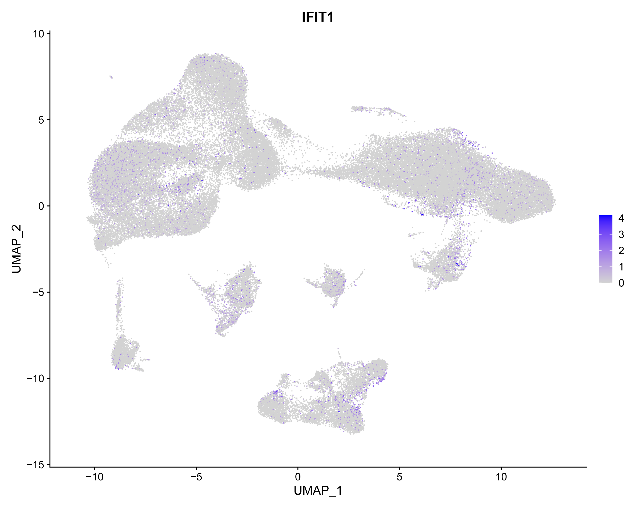


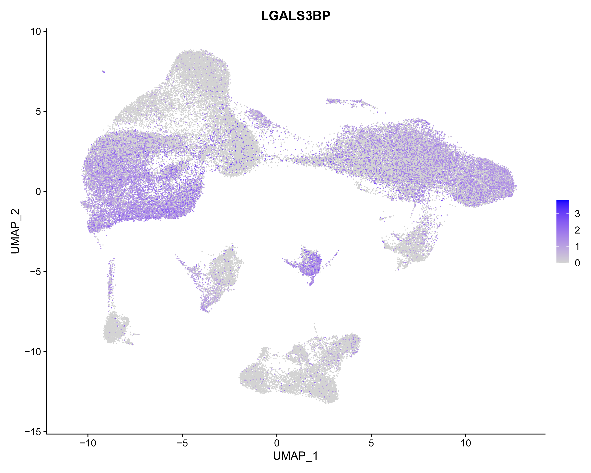

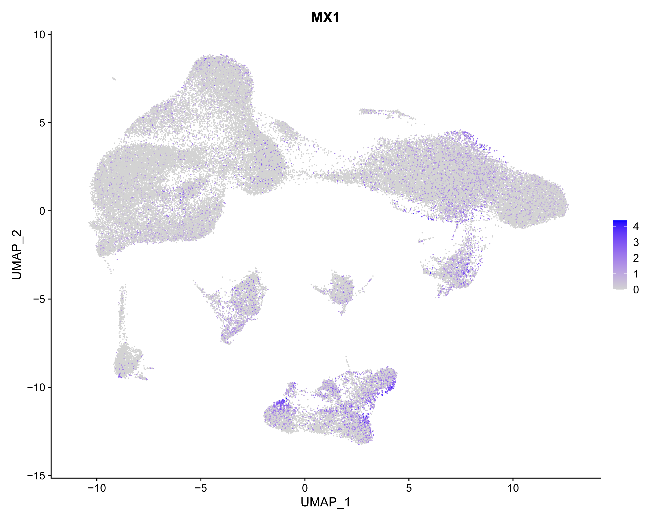


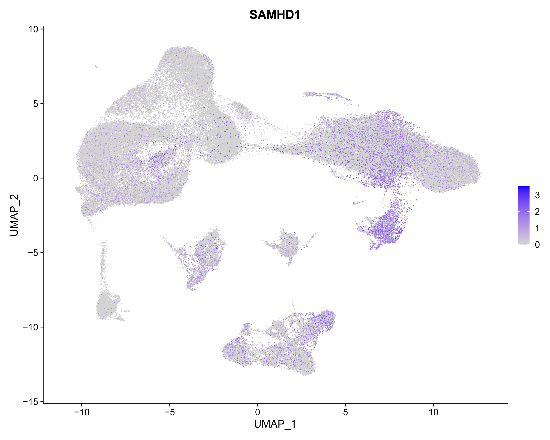

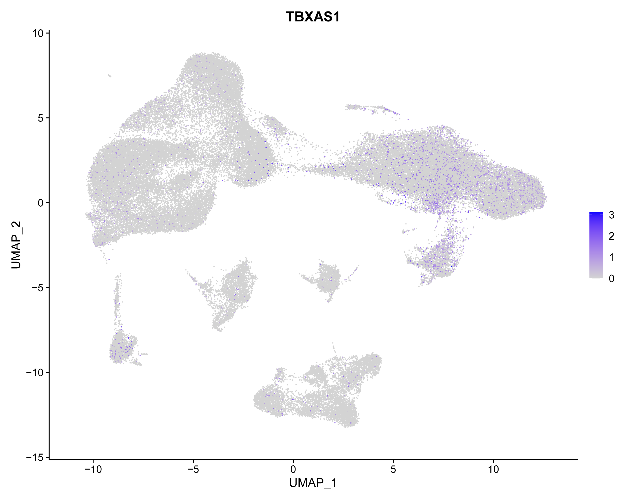


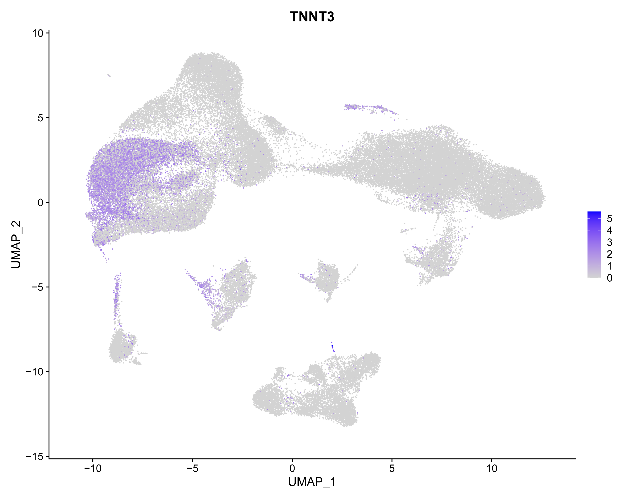


**Fig. S12.** Expression of APOL1, ALDH1A1, CNN2, FLNA, IFI44L,

IFIT1, LGALS3BP, MX1, SAMHD1, TBXAS1, and TNNT3 in UPS clusters.


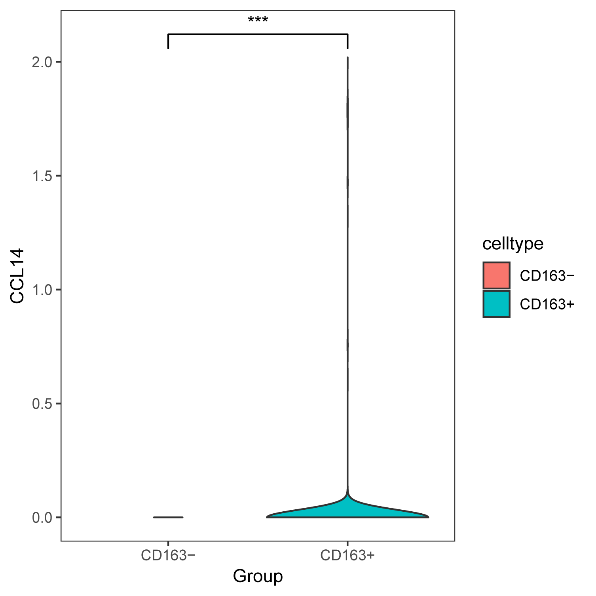

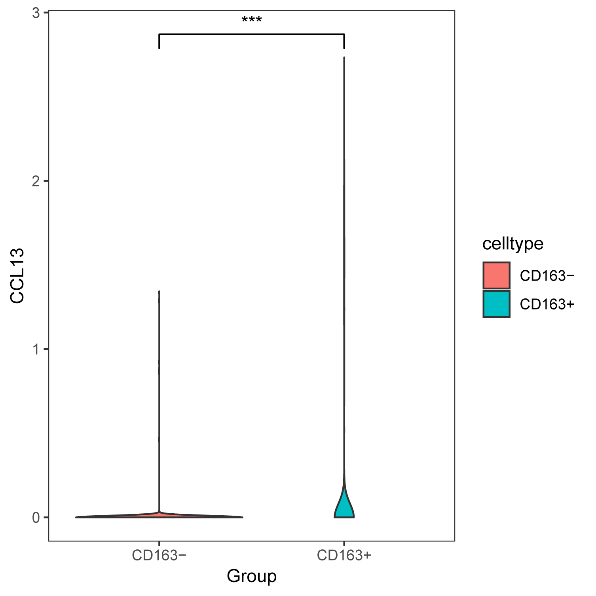


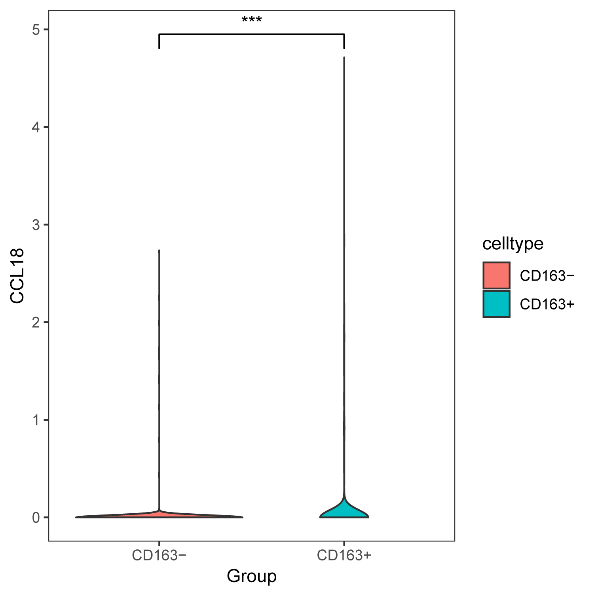

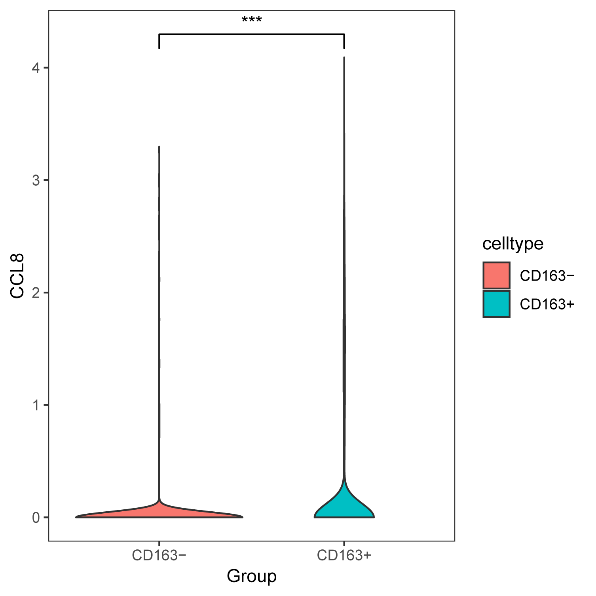


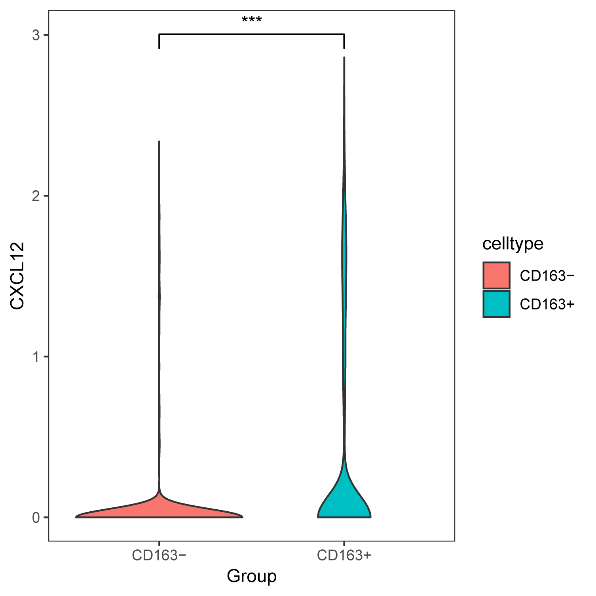


**Fig. S13.** Chemokines (CCL14, CCL13, CCL18, CCL8, and CXCL12)

were significantly upregulated in CD163+ macrophages compared with that in

CD163- macrophages.


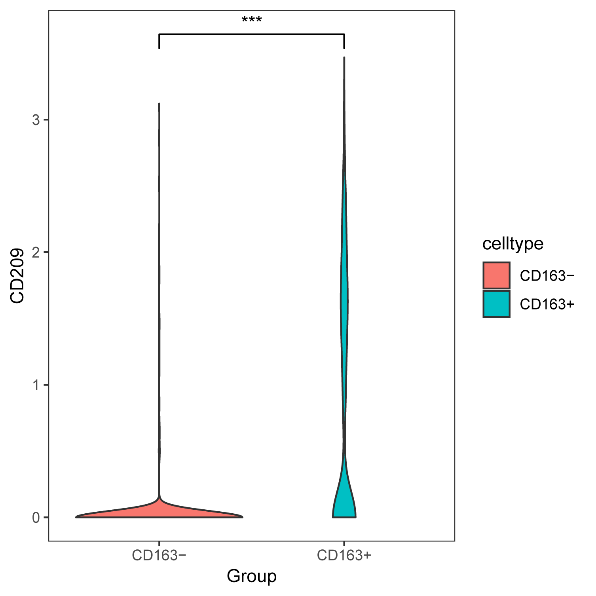

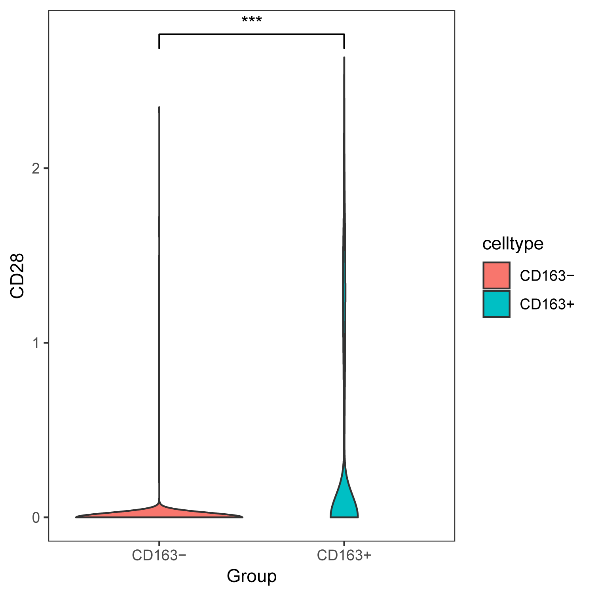


**Fig. S14.** Phagocytosis-associated molecular CD209 and co-stimulation

factor CD28 showed abundance in CD163+ macrophages compared with that in

CD163- macrophages.


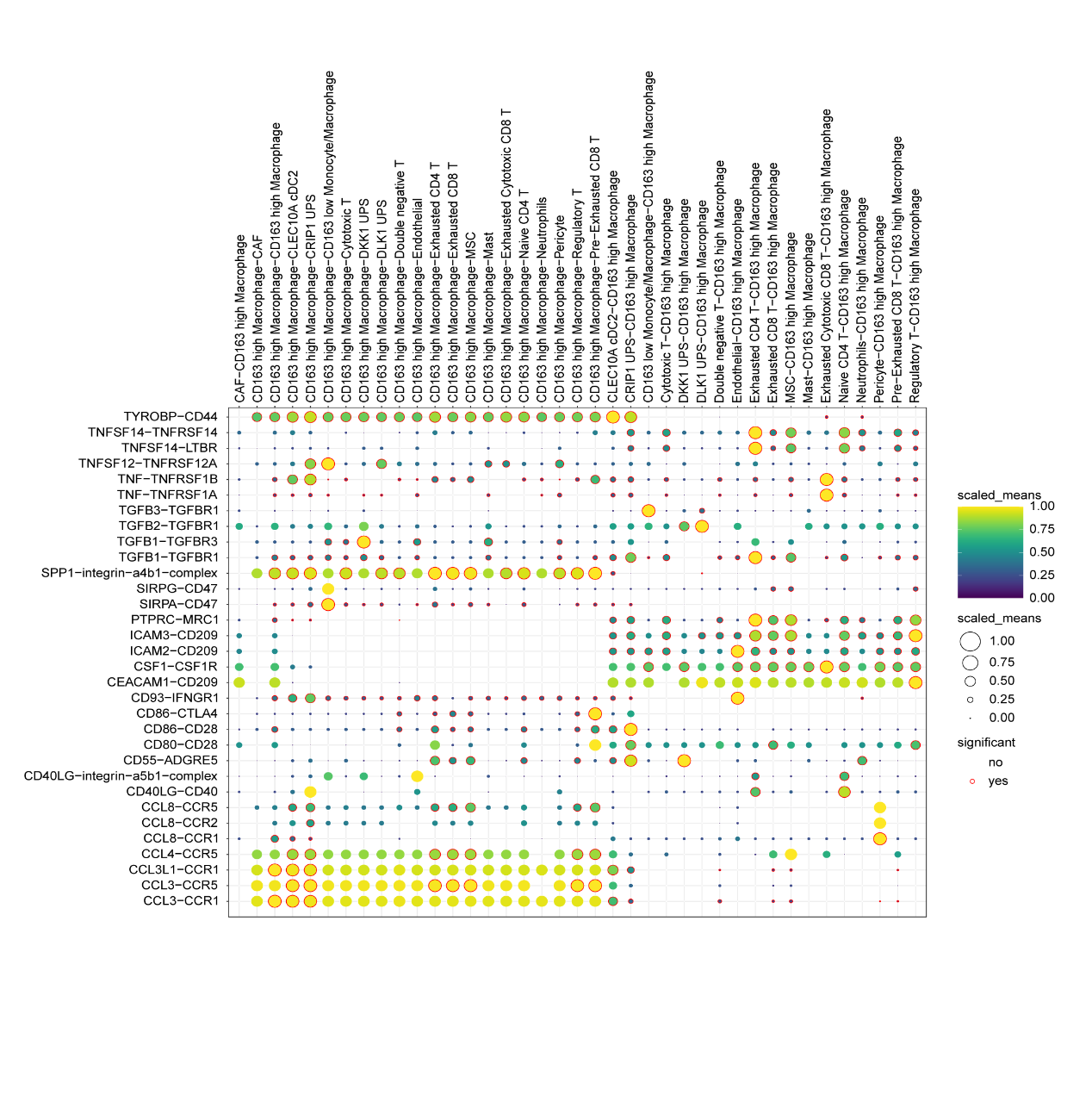


**Fig. S15.** The representative interaction network between CD163+(high) macrophages and other cells.
